# A xylan glucuronosyltransferase gene exhibits pleiotropic effects on cellular composition and leaf development in rice

**DOI:** 10.1038/s41598-020-60593-3

**Published:** 2020-02-28

**Authors:** Dawei Gao, Wenqiang Sun, Dianwen Wang, Hualin Dong, Ran Zhang, Sibin Yu

**Affiliations:** 1 National Key Laboratory of Crop Genetic Improvement, College of Plant Science and Technology, Huazhong Agricultural University, Wuhan, 430070 China; 2 Biomass & Bioenergy Research Centre, Huazhong Agricultural University, Wuhan, 430070 China

**Keywords:** Gene expression, Plant breeding

## Abstract

Leaf chlorophyll content is an important physiological indicator of plant growth, metabolism and nutritional status, and it is highly correlated with leaf nitrogen content and photosynthesis. In this study, we report the cloning and identification of a xylan glucuronosyltransferase gene (*OsGUX1*) that affects relative chlorophyll content in rice leaf. Using a set of chromosomal segment substitution lines derived from a cross of wild rice accession ACC10 and *indica* variety Zhenshan 97 (ZS97), we identified numerous quantitative trait loci for relative chlorophyll content. One major locus of them for relative chlorophyll content was mapped to a 10.3-kb region that contains *OsGUX1*. The allele *OsGUX1*^*AC*^ from ACC10 significantly decreases nitrogen content and chlorophyll content of leaf compared with *OsGUX1*^*ZS*^ from ZS97. The overexpression of *OsGUX1* reduced chlorophyll content, and the suppression of this gene increased chlorophyll content of rice leaf. OsGUX1 is located in Golgi apparatus, and highly expressed in seedling leaf and the tissues in which primary cell wall synthesis occurring. Our experimental data indicate that *OsGUX1* is responsible for addition of glucuronic acid residues onto xylan and participates in accumulation of cellulose and hemicellulose in the cell wall deposition, thus thickening the primary cell wall of mesophyll cells, which might lead to reduced chlorophyll content in rice leaf. These findings provide insights into the association of cell wall components with leaf nitrogen content in rice.

## Introduction

Rice (*Oryza sativa*. L) providing a staple food for more than half of the population in the world, is a monocot model species for genetics, biology and functional genomics studies. Leaf photosynthesis has great potential for the improvement of rice yield, which will significantly contribute to addressing food demand challenge. The chloroplast of green tissue or leaf, as the most important supporter of carbon fixation and energy transformation, plays important roles in photosynthesis^1–3^. Both genetic and environmental factors have effect on the biochemical composition in chloroplast, thus affecting photosynthetic rate^4^.

Previous studies have indicated that leaf color is a sensitive indicator of crop growth, metabolism and nutritional status, and it is closely related to the content of photosynthetic pigment, and positively correlated with leaf nitrogen (N) content^5–7^. Understanding the genetic and physiological bases of leaf nitrogen status is essential for efficient crop production and nitrogen management in intensive rice cropping systems. Leaf greenness is determined by specific properties, such as leaf chlorophyll content and chloroplast development, and leaf morphological characteristics (leaf thickness, surface structure and wall components). To study the genetic and molecular basis of leaf color, more than one hundred of rice mutants associated with leaf colors and chlorophyll content have been identified (http://archive.gramene.org/db/genes/). Several genes have been implicated in the chlorophyll biosynthesis, degradation, and regulation of chloroplast development through analyses of chlorophyll-deficiency mutations, which are typically reflected in leaf color^8–11^. For example, *SGR* (*Stay Green Rice*) gene encodes an ancient protein containing a putative chloroplast transit peptide. The *sgr* mutant was characterized by chlorophyll retention with stable chlorophyll-protein complexes and thylakoid membrane structures. Overexpression of *SGR* reduced the number of thylakoid lamellae in the chloroplasts and the chlorophyll content in growing leaves^12^. However, there have been few reports on cloning of the gene influencing leaf color or leaf N content in natural variation populations in rice. Despite a few studies on the effect of carbohydrate metabolism on chloroplast development and plant development^13–16^, it is still largely lacking of the genetic connection of the leaf color and the cellular components in leaf primary cell walls. The knowledge of the genes related to cellular components that influence chlorophyll content and leaf structure is limited.

Rice is a grass where xylan is the major non-cellulosic component of the leaf primary cell walls. Many glucuronyltransferase genes of Glycosyltransferase Family 8 (GT8) are reported responsible for sugar substitutions on the xylan of the primary cell walls. GT8 members are involved in several unique types of glycoconjugate and glycan biosynthetic processes and include the galacturonosyltransferase (GAUT) and GAUT-Like clade, galactinol synthase (GolS), inositol phosphorylceramide glucuronosyltransferase1 (IPUT1), and glucuronic acid substitution of xylan (GUX) protein clades with distinct functions in plants^17,18^. Particularly, *Arabidopsis* GUX1, GUX2 and GUX3 make different distinct patterns of glucuronic acid (GlcA) and/or methylglucuronic acid (MeGlcA) substitutions^19,20^. GUX1 and GUX2, both required for substitution of the xylan backbone with [Me]GlcA, are related to secondary wall biosynthesis. The mutants *gux1* and *gux2*, lose the xylan glucuronyltransferase activity, affecting the addition of GlcA or MeGlcA to xylan^17^. Such an addition influences normal secondary wall deposition and plant development. GUX3 is involved in addition of the GlcA decorations on xylan of the primary cell wall in *Arabidopsis*^20^. Further, the GUX1/2/3 proteins exhibit capability of transferring GlcA residues from the UDP-GlcA donor onto xylooligomer acceptors in tobacco BY2 cells^21^. The *gux1/2/3* triple mutant has been reported to cause a complete loss of GlcA and MeGlcA side chains on xylan. However, dissimilar phenotype in plant growth was observed for the *gux1/2/3* triple mutant in different studies^20,21^. One study showed that the triple mutant reduced secondary wall thickening, collapsed vessel morphology and inhibited plant growth^21^. Another thorough study revealed that the absence of GlcA xylan in cell wall had no impact on plant growth in the *gux1/2/3* triple mutant^20^. Further investigation of this discrepancy is required. Therefore, it is very interested in whether the rice homologous genes of *GUXs* associated with cellular components affect plant growth in rice.

In the present study, a set of chromosomal segment substitution lines (CSSL) derived from a wild rice accession (designated as ACC10) and an *indica* cv. Zhenshan 97B (ZS97) was used for detecting quantitative trait loci (QTL) for relative chlorophyll content and nitrogen content in rice leaf. Using a CSSL-derived F_2_ population, a major QTL for leaf chlorophyll content measured by Soil-Plant Analysis Development (SPAD) value was finely mapped to a 10.3-kb region. Transgenic experiments revealed that *OsGUX1* affects primary cell wall components corresponding to the QTL for relative chlorophyll content.

## Results

### Fine mapping of *qNC1.2* for leaf nitrogen content in CSSLs

As a first step to identify the key genes underlying QTLs for leaf color or nitrogen content, a set of chromosome segment substitution lines, each of which carries a single or few chromosome segments of a wild rice accession (ACC10) in the ZS97 genetic background were developed. To determine the genotype precisely, the CSSLs were re-analyzed using a SNP chip RICE6K (Illumina) containing 5,102 SNPs markers with an average density of 12 SNPs per Mb^22^. The results display that each line contained one or a few introduced donor segments at a particular chromosomal region against the uniform genetic background of ZS97, and all the substitution segments together covered the entire ACC10 genome, except one gap on chromosome 7 physical region (22.34–24.39 Mb). Genotyping of the CSSL population composed of 111 lines was defined as a total of 379 bins (B1 to B379) across the whole genome (Fig. S2). The physical length of the bins ranged from 19.7 kb to 8.5 Mb with a median size of 0.69 Mb.

Two parental lines (ZS97 and ACC10) differed markedly in relative chlorophyll content and nitrogen content in flag leaf which were determined by SPAD-value (Table S1). ZS97 had higher relative chlorophyll content, which was significantly different from ACC10. The CSSL population varied widely in relative chlorophyll content. Most lines had phenotypic values similar to that of ZS97, whereas several lines exhibited significantly differences in relative chlorophyll content, compared with ZS97. Four putative QTLs for relative chlorophyll content were identified in QTL analysis of the population with 379 bin markers (Table S2). Of these 4 QTLs, a major locus *qNC1.2* located on the long arm of chromosome 1 exhibited the largest effect, explaining 26.9% of the phenotypic variation in the population.

**Figure 1:**
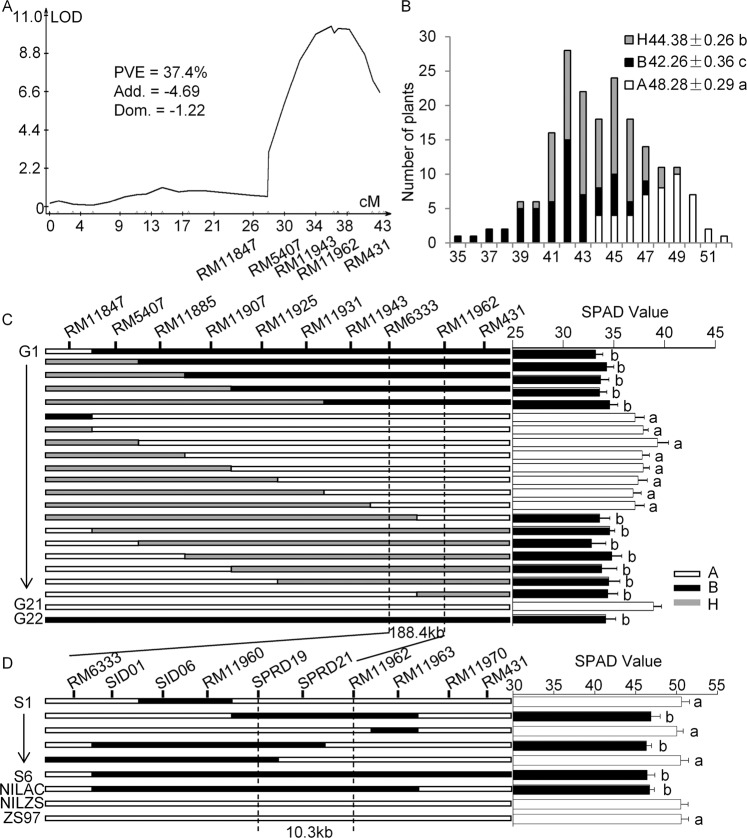
Detection and fine mapping of *qNC1.2* for leaf nitrogen content in rice. (**A**) Confirmation of the QTL for SPAD value using a CSSL-derived F_2_ population comprised of 190 individuals. PVE% represents the phenotypic variance explained by the QTL; Add and Dom represent the additive and dominate effect, respectively. (**B**) Distribution of three genotypes in the population assayed by marker RM11943 that was tightly linked with the QTL. (**C**) Progeny testing of the recombinants delimited the QTL into a 188.4-kb interval. (**D**) Finely mapping of *qNC1.2* to a 10.3-kb region between RM6333 and RM11962. At right histogram, progeny testing of the recombinant lines with average SPAD value in flag leaves are indicated. Each bar represents mean ± SE (n = 16). Different letters represent a significant difference at P < 0.01 by Student’s t-test between each recombinant line and the control NIL^ZS^. Genotypes A, B, and H denote ZS97, ACC10, and heterozygote, respectively.

To validate the effect of *qNC1.2* on relative chlorophyll content or nitrogen content, we constructed a CSSL-derived F_2_ segregating population at the target QTL and genotyped the population by using 16 polymorphic SSR markers within the target region. QTL analysis of a small CSSL-derived F_2_ population consisting of 190 individuals indicated that *qNC1.2* for relative chlorophyll content in flag leaf was flanked by RM11847-RM431, explaining 37.4% of the phenotypic variation (Fig. 1A). Three genotypes at RM11943 tightly linked with *qNC1.2* displayed significant differences in SPAD-value (Fig. 1B). The ACC10 alleles decreased relative chlorophyll content in a partial dominant way. Ninety-five recombinants within RM11847-RM431 were genotyped with additional ten SSR markers. From them, 20 representative recombinants with the wild rice introduced fragments overlapping the QTL region were selected for progeny testing. The progeny testing delimited the QTL into a 188.4-kb region between RM6333 and RM11962 (Fig. 1C).

**Figure 2:**
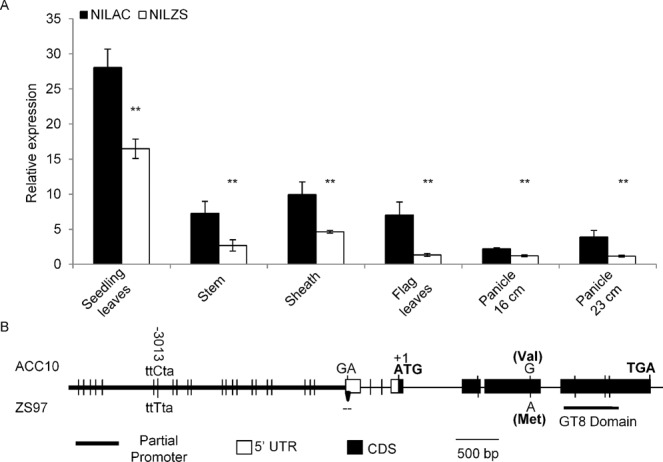
Expression profile and sequence variation of *OsGUX1* in the near isogenic lines (NILs). (**A**) qRT-PCR analysis of the expression of *OsGUX1* relative to the geometric mean of three control genes (*Ubiquitin*, *Actin1*, *β-tubulin*) in various tissues from NIL^AC^ and NIL^ZS^. The experiment was conducted in biological triplicates with four technical replicates. Data are presented as means ± SE (n = 3). The asterisks indicate significant differences between NIL^AC^ and NIL^ZS^ by student’s t-test (*P < 0.05, **P < 0.01). (**B,C**) Schematic of the gene model showing allelic variation of *OsGUX1* between ACC10 and ZS97. Arrow represents nucleotides deletion in the promoter region. Vertical line at a certain position indicates single nucleotide polymorphism between ACC10 and ZS97.

To finely map *qNC1.2*, we further screened a total of 1200 individuals from the F_2_ population using the markers RM6333 and RM431 (Fig. 1C). Seven new recombinants were obtained from RM6333-RM431 region by using ten additional SNP or InDel markers. Their homozygous lines were developed and used for the phenotypic investigation. During the process, two near-isogenic lines (NIL^ZS^ and NIL^AC^) containing the contrasting alleles (ZS97 and ACC10) within an approximately 200-kb region of *qNC1.2* were developed. The analyses of these homozygous recombinant lines delimited *qNC1.2* to a 10.3-kb region between markers SPRD19 and RM11962 (Fig. 1D). This small region contained two predicted genes (LOC_Os01g65770 and LOC_Os01g65780) in the genome database (http://rice.plantbiology.msu.edu/cgi-bin/gbrowse). The former (LOC_Os01g65770) encoded a putative express protein and it was hardly expressed in any tissues, while the latter (LOC_Os01g65780) encoded a putative plant glycosyltransferase and was constitutively expressed at various tissues (http://crep.ncpgr.cn/). Sequence analyses revealed that large variations (30 SNPs and InDels) occurred in the promoter and coding region of LOC_Os01g65780 of ACC10 and ZS97. In addition, the relative expression of this gene in several tissues, such as seedling leaf, stem, sheath, flag leaf, and panicle of NIL^AC^ was significantly higher than that of NIL^ZS^ (Figs. 2A, S3). Hence, LOC_Os01g65780 was the most likely candidate gene underlying *qNC1.2*. Since LOC_Os01g65780, closest to GUX1 and GUX3 in *Arabidopsis*, displayed the conserved GT8 motif (Fig. S4A), we hereafter referred to it as *OsGUX1*.

### *OsGUX1* underlying *qNC1.2* for chlorophyll content in leaf

To determine whether *OsGUX1* affects leaf development, we constructed transgenic lines overexpressing *OsGUX1* of ACC10 (OE780^AC^) in the ZS97 background. The transcript levels of the gene in three independent positive OE780^AC^ lines were approximately 100–450 folds higher than those in the negative transgenic lines (Fig. S5A). The SPAD-value, chlorophyll *b* content, and nitrogen content in flag leaves of positive OE780^AC^ lines were significantly lower than those of the negative lines (Fig. S5B–D).

**Figure 3:**
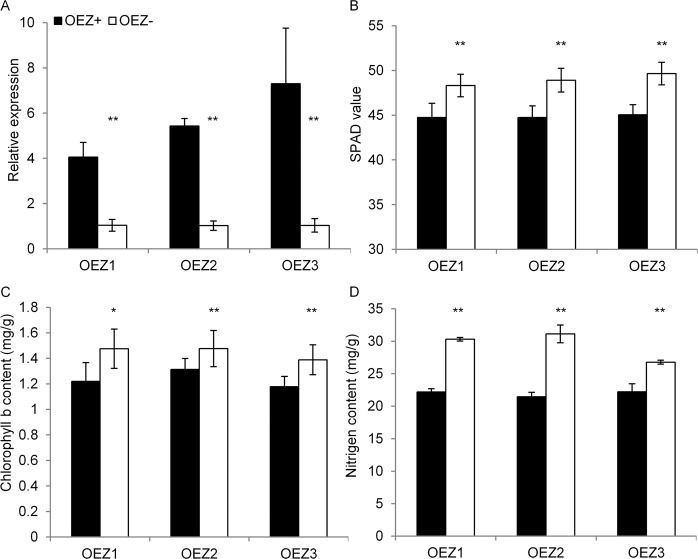
Relative expression and SPAD-related phenotype of flag leaves in three pairs of *OsGUX1*^*ZS*^ overexpression lines (OEZ). (**A**) Relative expression of *OsGUX1*^*ZS*^. Data represent the mean ± SE (n = 3). (**B**) SPAD value. (**C**) Chlorophyll *b* content. (**D**) Nitrogen content. Asterisks indicate significant differences between the positive transgenic lines (OEZ+) and negative lines (OEZ−) by student’s t-test (*P < 0.05, **P < 0.01). Data represent the mean ± SE (n = 8 biological repeats) except that the mean of leaf nitrogen content is derived from three repeats.

**Figure 4:**
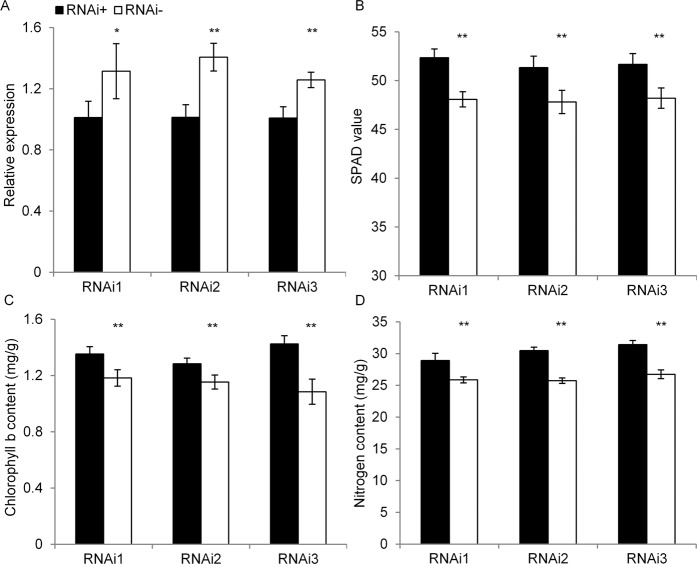
Relative expression and SPAD-related phenotype of flag leaves in three pairs of *OsGUX1*^*ZS*^ RNAi lines (RNAi 1–3). (**A**) Relative expression of *OsGUX1*^*ZS*^. (**B**) SPAD value. (**C**) Content of chlorophyll *b*. (**D**) Content of nitrogen. Asterisks indicate significant differences between the positive lines (RNAi+, black) and negative lines (RNAi−, white) by student’s t-test (*P < 0.05, **P < 0.01). The errors bar in (**B**,**C**) represent the mean ± SE (n = 8), data in (**A**,**D**) is derived from three repeats.

Sequence analyses revealed that only one nucleotide variation was observed in the fourth exon of the candidate gene between the parental lines, leading to an amino acid change (V267M) at the 267^th^ site with valine (V) in ACC10 and methionine (M) in ZS97 (Fig. 2B). To investigate whether the variant V267M affects chlorophyll content, we isolated *OsGUX1* from ZS97 to construct additional overexpression lines (OE780^ZS^). The transcript levels of the gene in three independent positive OE780^ZS^ lines were approximately 4–7 folds higher than those in the negative lines (Fig. 3A). Like OE780^AC^ lines did, the OE780^ZS^ lines decreased the SPAD-value, nitrogen content, and chlorophyll *b* content in leaves compared with those in the corresponding negative lines (Fig. 3B–D). In contrast, the down-regulation of *OsGUX1* expression in the positive RNAi lines (R780^ZS^) in the ZS97 background led to the significant increase in SPAD-value, chlorophyll *b* and nitrogen content at flowering stage (Fig. 4A–D). These results indicate that functional differences between the ACC10 and ZS97 alleles might not be due to the single structural variation in encoding amino acids, but rather to the transcriptional change. Additionally, the SPAD-value and chlorophyll content were decreased in OE780^ZS^ but they were increased in R780^ZS^ at seedling stage (Fig. S6), which was consistent with their variation tendency observed at flowering stage. Taken together, these results supports that *OsGUX1* is responsible for *qNC1.2*.

### Phylogenetic analysis and subcellular localization of OsGUX1

Using the predicted amino acids encoded by *OsGUX1*, we retrieved 9 homologous protein sequences in *Oryza sativa* and 16 homologs in *Arabidopsis thaliana*. Phylogenetic analysis revealed that these homologous proteins fell into three clades, namely, GolS, GUXs, and IPUT1, respectively (Fig. S4A). OsGUX1 was grouped into the GUXs and was closest to GUX1 and GUX3 in *Arabidopsis* (Fig. S4A), which contained the conserved amino acid sequences including the DxD and HxxGxxKPW motifs identified previously^23^. These results indicate that *OsGUX1* may have a similar function of *GUX1* and *GUX3* on cell wall components.

Subcellular localization analysis of the OsGUX1 fused with GFP revealed that OsGUX1 was localized to the Golgi apparatus in which xylan biosynthesis occurred. The single amino acid change (V267M) of OsGUX1 did not affect its localization in Golgi (Fig. S4B,C), which coincided with the localization of the homologous GUX1 and GUX2 that were previously reported as Golgi-localized glycosyltransferases in *Arabidopsis*^17,24^.

### *OsGUX1* affects cellular components and plant growth

**Figure 5:**
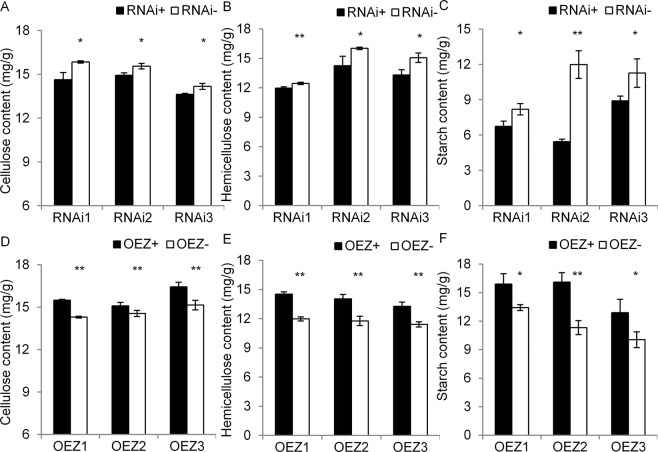
Cell components of flag leaves in *OsGUX1*^*ZS*^ RNAi and overexpression (OEZ) lines. (**A**,**D**) Cellulose content. (**B**,**E**) Hemicellulose content. (**C**,**F**) Starch content. Data represent the mean ± SE (n = 3, with eight leaf samples mixed for each repeat). The asterisks indicate significant differences between the positive (+) and negative (−) lines by student’s t-test (*P < 0.05, **P < 0.01).

Considering the effect of homologous genes *GUX1* and *GUX3* on cellular components in *Arabidopsis*^19–21^, we investigated several cellular components in the transgenic lines to determine whether *OsGUX1* affected cellular components and plant development in rice. Positive R780^ZS^ lines markedly reduced the content of cellulose, hemicellulose, and starch in flag leaf, compared with the negative lines (Fig. 5A–C). While positive OE780^ZS^ lines significantly increased the content of cellulose, hemicellulose and starch in flag leaf (Fig. 5D–F).

**Figure 6:**
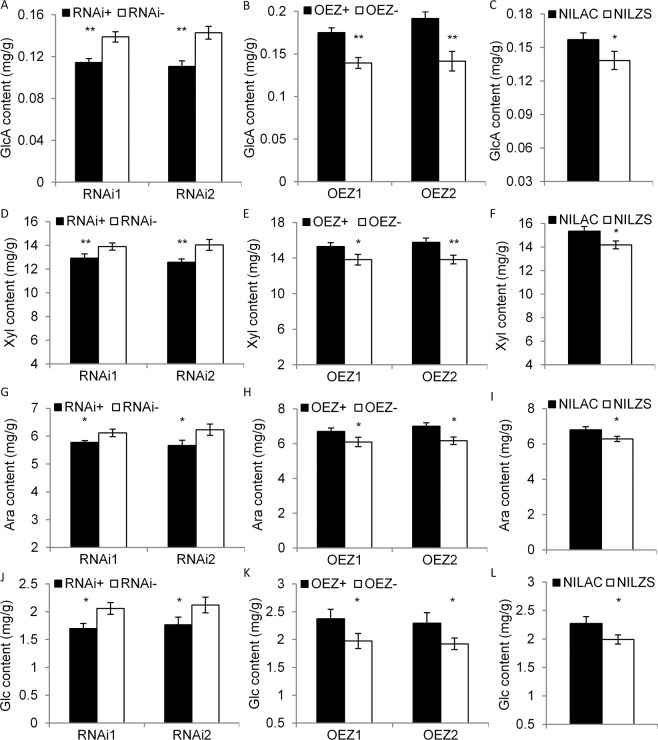
Monosaccharide analysis of flag leaves in *OsGUX1*^*ZS*^ RNAi, overexpression (OEZ) lines and NILs. (**A-C**) Glucuronic acid (GlcA). (**D**–**F**) Xylose (Xyl). (**G**–**I**) Arabinose (Ara). (**J–L**) Glucose (Glc). The asterisks indicate significant differences between the pairwise positive (+) and negative (−) lines and in NILs by student’s t-test (*P < 0.05, **P < 0.01). Data represent the mean ± SE (n = 4, with mixed eight leaf samples per repeat).

To further investigate whether *OsGUX1* affected sugar substitutions on the xylan in rice leaf, we also used GC-MS (Gas Chromatography-mass Spectrometer) analysis to determine glucuronic acid (GlcA) and several monosaccharides including xylose, arabinose, and glucose in flag leaves of the *OsGUX1* transgenic lines and NILs. The R780^ZS^ lines repressing *OsGUX1* resulted in a significant decrease in GlcA, xylose, arabinose, and glucose content (Fig. 6A,D,G,J). Whereas the OE780^ZS^ lines overexpressing *OsGUX1* caused an significant increase in GlcA and the three monosaccharides compared with the corresponding negative lines (Fig. 6B,E,H,K). The increased GlcA indicate that *OsGUX1* has the function of adding glucuronic acid residues onto xylan. The increased xylose, arabinose and glucose suggest that xylan synthesis and hemicellulose linked glucan may be affected. These results indicate that *OsGUX1* is involved in the xylan substitutions, leading to alteration of the cell wall depositions on rice leaf.

**Figure 7:**
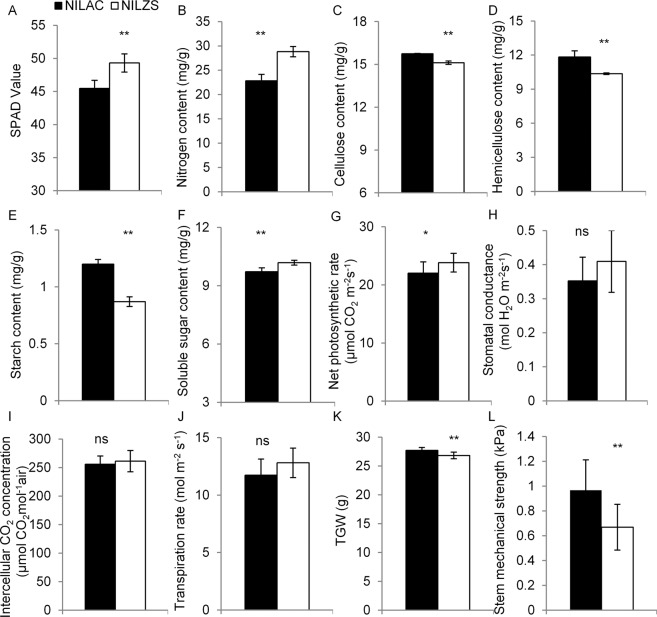
Phenotype investigation in NIL^AC^ and NIL^ZS^ on day 7 after heading. (**A**) SPAD value. (**B**) Nitrogen content. (**C**) Cellulose content. (**D**) Hemicellulose content. (**E**) Starch content. (**F**) Soluble sugar content. (**G**) Net photosynthetic rate. (**H**) Stomatal conductance. (**I**) Intercellular CO_2_ concentration. (**J**) Transpiration rate. (**K**) Thousand grain weight. (**L**) Mechanical strength of stems. The asterisks indicate significant differences between NIL^AC^ and NIL^ZS^ by student’s t-test (*P < 0.05, **P < 0.01, ns, non significant). Data represent the mean ± SE (n = 3, with at least eight leaf samples mixed per repeat).

In consistent with transgenic experiment results, NIL^AC^ significantly decreased leaf nitrogen content (Fig. 7A,B), but significantly increased the contents of hemicellulose, cellulose, and starch compared with NIL^ZS^ (Fig. 7C,D,E). The amounts of GlcA and the three monosaccharides in NIL^AC^ was also significantly higher than that of NIL^ZS^ (Fig. 6C,F,I,L). Notably, R780^ZS^ lines reduced seedling height and fresh weight (Fig. S7A,B). However, OE780^ZS^ lines promoted seedling growth (Fig. S7C,D). Consistently, the seedling weight and seedling height in NIL^AC^ were significantly higher than those in NIL^ZS^ (Fig. S8). In addition, NIL^AC^ displayed an increased thousand grain weight and enhanced mechanical strength of the stems compared with NIL^ZS^ (Fig. 7K,L). These results suggest that *OsGUX1* participates in accumulation of hemicellulose and cellulose, having strong effects on plant growth across various stages.

### Stable difference in leaf nitrogen content between NILs

We conducted a dynamic investigation of leaf nitrogen content in the NILs at different time points from day 6 before heading to 13 day after heading (DAH) (Fig. S9A). A peak of SPAD-value appeared on 7 DAH, the value began to decline gradually from 7 DAH. The net photosynthetic rate of NIL^AC^ significantly reduced, compared with that of NIL^ZS^ (Fig. 7G), which was consistent with the observation of an decreased total chlorophyll content in flag leaves. However, no difference in the stomatal conductance, intercellular CO_2_ concentration and transpiration rate was observed between NIL^AC^ and NIL^ZS^ at heading stage (Fig. 7H–J).

To determine whether the expression level of *OsGUX1* was affected by nitrogen application, we conducted hydroponic experiments with NILs at seedling stage under three nitrogen gradients. The results revealed that relative expression level of *OsGUX1* was induced with the increase of nitrogen application in both NIL^AC^ and NIL^ZS^, and that the expression level of *OsGUX1* in NIL^AC^ was significantly higher than that in NIL^ZS^ under all three nitrogen gradients (Fig. S9B). Particularly, under low nitrogen level, the expression of *OsGUX1* in NIL^AC^ was almost two times as high as that in NIL^ZS^. However, the relative expression of *OsGUX1* was slightly inhibited under high nitrogen level in both NILs compared with that under normal nitrogen application. As a result, the SPAD-value, nitrogen content, and chlorophyll *b* in NIL^AC^ were significantly lower than those in NIL^ZS^ under all nitrogen levels (Fig. S9), although a significant increase in the three assayed traits were observed between low and normal nitrogen level for the same NIL. These results indicated that *OsGUX1* associated with the leaf N content was independent of exterior N application.

### Thickened primary cell wall may affect relative chlorophyll content

**Figure 8:**
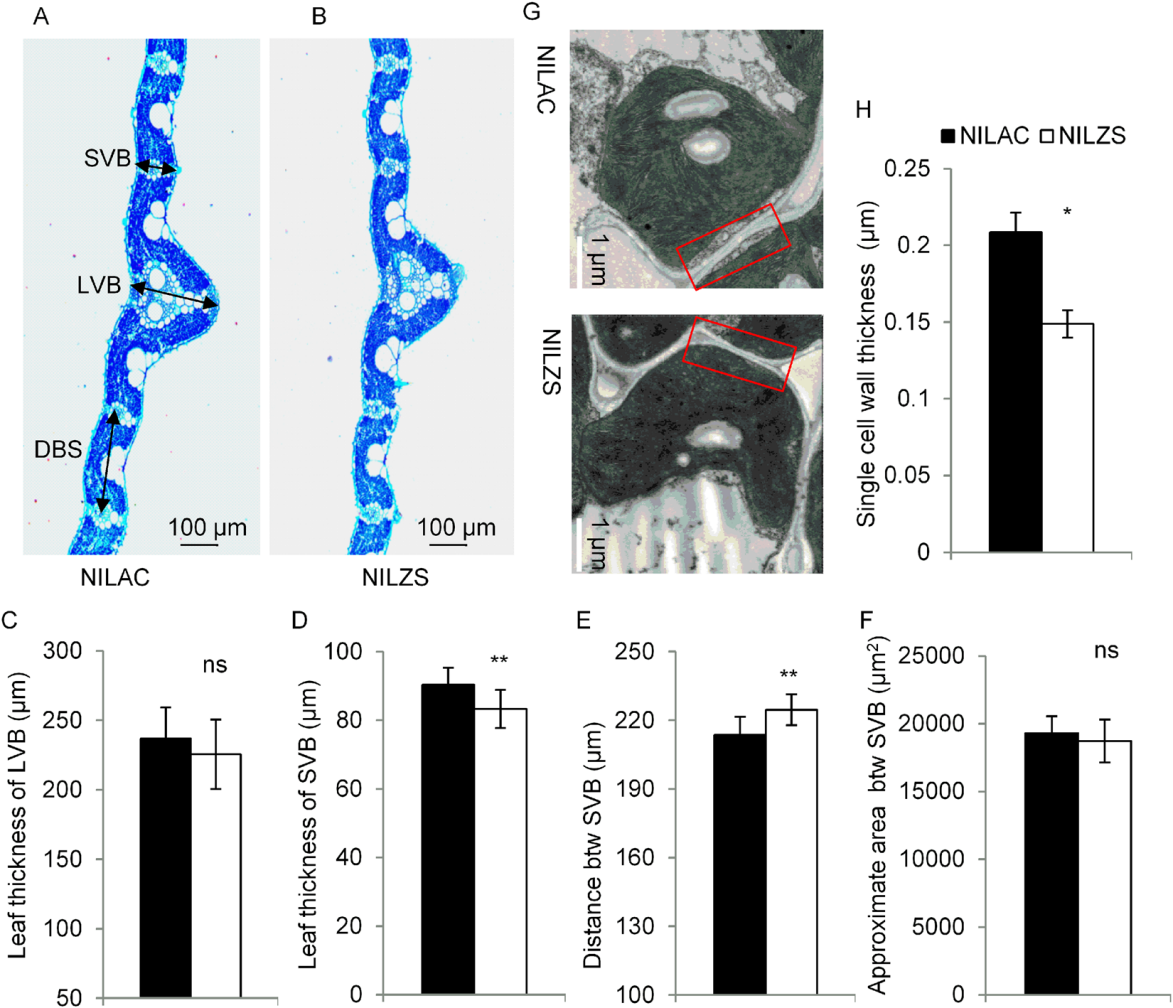
Leaf anatomical characteristics and cell wall thickness in NILs. (**A**,**B**) Cross-sections of flag leaves stained with toluidine blue, (**A**) NIL^AC^; (**B**) NIL^ZS^. (**C**,**D**) Comparison of thickness of flag leaves in the NILs. (**C**) Leaf thickness of LVB (large vascular bundle); (**D**) Leaf thickness of SVB (small vascular bundle). (**E**) Distance between adjacent SVBs. (**F**) Approximate area within adjacent SVBs (μm^2^). (**G**) Transmission electron microscopic observation of mesophyll cell walls in the NILs. (**H**) Cell wall thickness in the NILs. Data represent the mean ± SE (n = 5, with 15 leaf sections harvested from 5 plants for each replicate). The asterisks indicate significant differences between NIL^AC^ and NIL^ZS^ by student’s t-test (*P < 0.05, **P < 0.01).

It has been reported that the linear relationship between SPAD value and leaf N concentration varies with crop growth stage and variety, mostly due to leaf thickness^25^. Thus, we compared the size or area of large vascular bundle (LVB) and small vascular bundle (SVB) through cross-sections of flag leaves of NILs, which represented the thickness of leaves (Fig. 8A,B). The histological section analysis revealed no difference in the average size of LVB between NIL^AC^ and NIL^ZS^ (Fig. 8C), while the thickness of SVB of NIL^AC^ was significantly larger than that of NIL^ZS^ (Fig. 8D), but the length between adjacent SVBs of NIL^AC^ was shorter than that of NIL^ZS^ (Fig. 8E). Consequently, no difference in the cross-sectional area containing mesophyll cells was observed between NILs (Fig. 8F). Intriguingly, the observation of the mesophyll cells by a transmission electron microscope revealed that the primary cell wall of mesophyll cells in NIL^AC^ was significantly thicker than that in NIL^ZS^ (Fig. 8G,H). These results suggest that primary cell wall of mesophyll cells might affect relative chlorophyll content in leaf.

### Expression of xylan and cellulose biosynthesis-related genes

Because cellulose and hemicellulose contents were increased in the overexpression *OsGUX1* transgenic lines and NIL^AC^, we used quantitative RT-PCR to analyze transcript levels of several key genes related to the wall polymers syntheses to explore whether *OsGUX1* affects xylan and cellulose syntheses. The results reveal that the transcript levels of eight biosynthetic genes involved xylan synthesis (*OsIRX9L* and *OsIRX14*) and cellulose synthases (*OsCesA1*, *OsCesA2*, *OsCesA3*, *OsCesA5*, *OsCesA6*, *OsCesA8*) in NIL^AC^ were significantly higher than that of NIL^ZS^ (Fig. S10A,B). It has been reported that these cellulose-related genes are strongly co-expressed in seedlings and other typical young vegetative tissues rich in primary cell walls^26^, and that *OsIRX9L* and *OsIRX14* are involved in the construction of xylan backbones in primary and secondary cell walls^27^. The elevated expression of xylan synthesis-related genes and cellulose synthase genes in NIL^AC^ was consistent with the increased cellulose, hemicellulose levels in leaves described above. These results further suggest that *OsGUX1* participates in the processes of xylan and cellulose biosynthesis.

Interestingly, lower soluble sugar content in NIL^AC^ was observed than those in NIL^ZS^ (Fig. 7F). We then analyzed the expression levels of several sucrose-related genes and UDP-Glc in flag leaves of both NILs at the 7 DAH. These genes have been reported to be involved in the carbon supply during cell wall synthesis^28^. The quantitative RT-PCR results indicate that the assayed sucrose-related genes were significantly induced in NIL^AC^, compared with those in NIL^ZS^ (Fig. S10C).

It is notable that *PHD1* (*Photoassimilate Defective 1*) was significantly repressed in NIL^AC^ compared with that in NIL^ZS^ (Fig. S10C). Previous study reported that *PHD1* encoding a chloroplast-localized UDP-Glc epimerase was responsible for the biosynthesis of UDP-galactose in chloroplast, and that *PHD1* played an important role in supplying sufficient galactolipids to thylakoid membranes for chloroplast function and photosynthetic activity^29^. The *phd1* mutant decreased chlorophyll content, photosynthetic activity, and altered chloroplast ultrastructure. Thus, *OsGUX1* might play a role in affecting the assignment of carbonhydrate sources and the increased use of UDP-Glc for the cell wall biosynthesis.

## Discussion

This present study reports the cloning and identification of the xylan glucuronosyltransferase gene *OsGUX1* that affects relative chlorophyll content in rice. Leaf chlorophyll content is an important physiological indicator of plant stress^30^, senescence^31^, and nitrogen content^32^. However, the genetic linkage between chlorophyll content and leaf structure remains largely unknown. As a first step toward dissecting the genetic bases of such complex trait, we used a SPAD chlorophyll meter to explore the key factors related to relative chlorophyll content in flag leaves in the developed CSSL population from the cross of ACC10 and ZS97 (Fig. S1). We have identified at least four loci for leaf chlorophyll content (Table S2). Of them, three loci are localized to the same or nearby regions for chlorophyll content as reported in previous studies in rice^33–39^. For example, *qNC1.1* in our study was found in the same region related to chlorophyll content reported in other studies^36,37^. *qNC7.1* was close to the QTL detected in the reports^38,39^. *qNC10.1* was mapped to the same location reported previously^36,40^. This co-localization supports the accuracy of the QTL analysis of leaf chlorophyll content using the CSSL population. As a major locus *qNC1.2* is a newly detected for leaf chlorophyll content, we thus using a map-based cloning strategy to identify the gene underlying *qNC1.2*, and have defined *OsGUX1* as the candidate gene for leaf chlorophyll content (Fig. 1). The allele *OsGUX1*^*AC*^ from a wild rice ACC10 significantly decreased nitrogen content and chlorophyll content of leaf, resulting in a reduction in photosynthetic rate of leaf compared with *OsGUX1*^*ZS*^. The transgenic lines overexpressing *OsGUX1* decreased relative chlorophyll content measured as SPAD-value, chlorophyll *a*, chlorophyll *b*, and carotenoid content in leaves at seedling stage (Fig. S6K–N), and also reduced the contents of chlorophyll in flag leaves at heading stage (Fig. 3B–D). While, the transgenic lines suppressing *OsGUX1* increased the contents of chlorophyll and nitrogen in leaves (Fig. 4B–D). It is notable that the expression patterns of *OsGUX2* and *OsGUX3*, two close homologous genes of *OsGUX1*, are different from that of *OsGUX1* (Figs. 2A and S10A,B). *OsGUX2* (LOC_Os02g35020.1) has high expression in flag leaves and low expression in stems, while *OsGUX3* (LOC_Os03g08600.1) has high expression in stems and low expression in leaves (Fig. S11). However, there are no significant differences in the expression of *OsGUX2* and *OsGUX3* in NIL^AC^ compared with that of NIL^ZS^. Furthermore, the expression of these two homologs was not altered in the *OsGUX1-*RNAi lines (Fig. S11C,D), suggesting that significant difference in leaf chlorophyll content in the *OsGUX1-*RNAi lines and NILs did not result from *OsGUX2* and *OsGUX3*. Collectively, overexpression and suppression of *OsGUX1* experiments and NIL assays indicate that *OsGUX1* expression level affects leaf chlorophyll content in rice. This identification of the major locus like *qNC1.2* provides an opportunity to elucidate the relationship of the cell wall properties and chlorophyll content of leaf.

Our experimental data support that *OsGUX1* plays a role in adding glucuronic acid residues onto xylan, which affecting hemicellulose and cellulose levels of the leaf primary cell wall in rice. Firstly, *OsGUX1* has low expression in stems and high expression in leaves (Figs. 2A and S3), suggesting that it is involved in primary wall biosynthesis. Secondly overexpression of *OsGUX1* lines and NIL^AC^ exhibited higher levels of GlcA, xylose, arabinose, and glucose in flag leaves than their counterparts. In contrast, suppression of *OsGUX1* lines revealed the opposite effect on GlcA and the three monosaccharides (Fig. 6). These results provide strong evidence that *OsGUX1* is involved in sugar substitutions on xylan. It is expected that the sugar substitutions on the primary cell wall xylan may influence the amount and structure of hemicelluloses, further affecting other cell wall components. Thirdly, our results have revealed that higher transcript levels of xylan synthesis-related genes and cellulose synthase genes were induced in overexpression *OsGUX1* lines, and lower transcript levels of these synthesis genes in suppression of *OsGUX1* lines than the corresponding negative lines (Fig. S10). Consistently, overexpression of *OsGUX1* increased the content of cellulose and hemicellulose, while suppression of *OsGUX1* decreased the content of cellulose and hemicellulose in rice leaf (Fig. 5). In addition, the contents of cellulose and hemicellulose in NIL^AC^ were significantly higher than those in NIL^ZS^. It has been reported that *GUX3* in *Arabidopsis*, a closely homologous gene to *OsGUX1* (Fig. S4), was highly expressed in the tissues in which primary cell wall synthesis occurring, and that *GUX3* is required for the addition of the GlcA decorations on the primary cell wall xylan and affected content of cellulose and hemicellulose^20,21^. Thus, the current data collectively suggest that *OsGUX1* has the function of transferring GlcA onto xylan, which affects the compositions of the primary cell wall in rice.

It is notable that *OsGUX1* could alter seedling growth and the mechanical strength of stems in rice (Figs. 7L and S7). In *Arabidopisis*, both *GUX1* and *GUX2* are responsible for the substitution of the xylan backbone with [Me]GlcA in the secondary cell wall^19,21,41^. The complete disappearance of secondary wall xylan GlcA and MeGlcA side chain in the triple mutant *gux1gux2gux3* had no impact on the xylem vessels and plant growth^20^. Hence, characterization of *OsGUX1* provides new knowledge about the role of GlcA substitutions in plant growth appear to be different from that in dicots.

The most noteworthy finding of the present study is that *OsGUX1* involves in xylan substitutions in the primary cell wall affecting leaf chlorophyll content. However, it is an open question how *OsGUX1* for xylan substitution plays an important role in leaf chlorophyll content. We propose that *OsGUX1* may affect cell wall compositions in the mesophyll cells, which indirectly influence the chlorophyll content. Our experimental data indicate that *OsGUX1* for the GlcA substitution of xylan in leaf has a strong effect on cell wall thickness of mesophyll cells, which may result in leaf development as evidenced by changes in nitrogen and chlorophyll. Several lines of evidence support this note. Firstly, a transmission electron microscope analysis revealed that the primary cell wall of mesophyll cells in NIL^AC^ was significantly thicker than that in NIL^ZS^ (Fig. 8G,H). Secondly, high expression of *OsGUX1* induced the expressions of xylan synthesis genes, which leads to the accumulation of cellulose and hemicelluloses (Figs. 7 and S10), thus thickening the primary cell walls. The increased cell wall thickness may be the additional contributor to mechanical strength (Fig. 7L). In addition, the thicker walls could cause decreased soluble sugars in leaf. In agreement with this case, lower soluble sugar level in NIL^AC^ was observed than that in NIL^ZS^ (Fig. 7F). As a response to the decrease of soluble sugar content, the expression levels of several genes related to the sucrose decomposition, transport, and metabolism in rice leaf were induced (Fig. S10C). It is also possible that the accumulation of cell wall components increased use of UDP-Glc, which may affect the UDP-galactose biosynthesis in chloroplast (Fig. S10C), leading to altered chloroplast ultrastructure and chlorophyll content. Therefore, *OsGUX1* thickened primary cell wall of mesophyll cells results in the decline in chlorophyll content of leaves, although the related genes participate in the chloroplast development process remains to be further investigated. Because chlorophyll content and nitrogen content of leaf is highly associated with photosynthesis and plant growth, discovery of the *OsGUX1* functions on primary cell wall components and leaf chlorophyll content will provide an option to improving the potential rice yield by adjusting leaf nitrogen status and enhancing the mechanical strength of stems under different nitrogen application conditions.

## Materials and Methods

### CSSL population and genotyping

The chromosomal segment substitution lines population was developed by a backcross scheme coupled with marker-aided selection. In this population, an *O. rufipogon* (accession: IRGC105491, ACC10) as donor parent was crossed with ZS97 as receptor parent, followed by six-time backcross (Fig. S1). A set of 111 lines, designated as CSSL1 to CSSL111, were used for phenotype collection and QTL detection (Fig. S2). The CSSL population was previously genotyped using 176 simple sequence repeat (SSR) markers on the published linkage map of rice (http://www.gramene.org). The results showed that each line harbored one or a few substituted ACC10 segments at particular chromosomal region in the genetic background of ZS97^42^. To determine the genotype precisely, a high-throughput Infinium RICE6K array (Illumina) containing 5102 SNP markers evenly distributed on the 12 rice chromosomes with an average density of 12 SNPs per Mb, was used to analyze the genotypes of CSSLs^22^.

The CSSL population, along with the parental lines ACC10 and ZS97, was grown in a three-row plot for each line with 10 individuals per row at a spacing of 16.7 cm × 26.6 cm in the experimental field of Huazhong Agricultural University (30.4°N, 114.2°E). The experiments followed a randomized block design with two replications for each line.

### Bin mapping of QTL

On basis of SNP genotyping, a bin was defined by a unique overlapping substitution segment from the CSSL population as described previously^43^. QTL analysis was conducted in the CSSL population with the bins as markers by the composite interval mapping method in the software IciMapping v4.0 (http://www.isbreeding.net/software/). QTL nomenclature followed the principle suggested by McCouch and CGSNL^44^.

### DNA extract and PCR reaction

DNA of the CSSLs and derived lines was extracted using the CTAB method^45^. The PCR reaction mixture consisted of 2 μl template (50 ng/μl), 2 μl 10× PCR Buffer (Mg^2+^ free), 1.6 μl dNTP (100 mM), 1 μl Forward and Reverse primers (10 mM), 0.12 μl rTaq (5 U/μl), and 13.28 μl ddH_2_O. The PCR reaction was performed at 94 °C for denaturation 5 min, and then for 35 cycles of 94 °C for 30 sec, 55 °C for 30 sec, followed by 72 °C for 5 min, and ended with 25 °C for 1 min.

### Data analysis in F_2_ population and progeny testing

QTL analyses in the CSSL-derived segregating population were performed using WinQTLcart 2.5^46^. Logarithm of the odds (LOD) score for declaring the presence of QTL for each trait was estimated by 1,000 permutations^47^. As a result of the permutation, an average threshold of 2.5 was used for a QTL declaration for each trait. Progeny testing of homozygous recombinant lines was conducted for precisely phenotyping. In the process of fine-mapping, two near isogenic lines (NILs) at the target QTL region were developed from a heterozygote of CSSL containing a small introduced target segment for the phenotypic characterization.

### RNA extract and quantitative real-time PCR analyses

Total RNA was isolated from the tissues using a RNA extraction kit (TRIzol reagent, Invitrogen) according to the manufacturer’s instructions. The cDNA was obtained from approximate 3 μg of total RNA using M-MLV First Strand Kit (Invitrogen, China). Quantitative RT-PCR was carried out in a total volume of 10 μl containing 4.6 µl of cDNA, 0.2 µM of primer mix, and 5 µl SYBR Green Master Mix (Roche) on a real-time PCR system StepOne^Plus^ (Applied Biosystems). Three rice genes *Ubiquitin* (LOC_Os03g13170), *Actin1* (LOC_Os03g50885) and *β-tubulin* (LOC_Os01g59150) were used as the internal control for all expression analyses. As similar expression results were observed regardless of which control genes used, a geometric mean of expressions of the three control genes was applied for the relative expression analyses for every assayed sample as described previously^48^. The assay was carried out with three biological replicates, and each replicate contained four technical repeats. The relative quantification of expression was based on the 2^−ΔΔ*C*T^ method^49^. All qRT-PCR primers are listed in the Supplementary Table S3. The sequences for all assayed genes in this study can be found in the Rice Genome Annotation Project website (http://rice.plantbiology.msu.edu/).

### Vector construction and rice transformation

To construct overexpression vectors for *OsGUX1*, the cDNA of *OsGUX1* from ZS97 and ACC10 was respectively amplified with the primers PU7804F and PU7804R, and cloned into T-vector (Promega). The sequence-confirmed clones containing *OsGUX1* were digested by *Sac* I and *Pst* I, and followed by the insertion of *OsGUX1* into the pCAMBIA1301S vector with the 35S promoter to produce the fusion vector 35S:*OsGUX1*.

To construct an RNAi vector for *OsGUX1*, a 403-bp fragment of *OsGUX1* was amplified from ZS97 cDNA with the primers PDS780F and PDS780R. And then this 403-bp fragment was inserted in the forward and reverse directions into pDS1301 vector, a modified version of pCAMBIA1301^50^. The overexpression vectors and RNAi suppression vectors were independently introduced into ZS97 by *Agrobacterium*-mediated transformation as described previously^51^. The transgenic rice plants were confirmed by PCR using specific primers (Table S3).

### Subcellular localization of OsGUX1 protein

For investigating the subcellular localization of the OsGUX1^AC^ and OsGUX1^ZS^ protein, the coding region of *OsGUX1*^*AC*^ and *OsGUX1*^*ZS*^ was cloned into the PM999-35S vector fused with C-terminal green fluorescent protein (GFP), respectively. The resultant construct (OsGUX1^AC^-GFP or OsGUX1^ZS^-GFP) and the Golgi marker Man1-RFP (red fluorescent protein) were transiently expressed in rice protoplasts as described previously^52,53^. The fluorescence signal was observed with a confocal laser scanning microscope (Leica FV1200, Olympus) after the transformed cells were incubated in darkness at 28 °C for 16–18 h, with excitation wavelengths of 488 nm and 561 nm for GFP and RFP, respectively.

### Protein sequence of multiple alignment

The protein sequences of OsGUX1^ZS^ and OsGUX1^AC^ were aligned with the homologous proteins that were retrieved from Phytozome v12.1 database^54^. The multiple sequence alignment of the full-length proteins was performed in the complete alignment method by the ClustalX in MEGA 6^55^. The unrooted phylogeny tree was developed using the neighbor-joining method in the software MEGA 6.

### Phenotypic measurements

Due to a unique linear relationship between Soil-Plant Analysis Development (SPAD) values and leaf nitrogen concentration level, a SPAD chlorophyll meter was used as a quick and nondestructive tool for estimating relative chlorophyll content of leaf^56^. SPAD values were taken from the fully extended flag leaf on the main stem around the heading stage using a SPAD meter (SPAD-502, Konica-Minolta, Japan). Ten readings taken from the side of the midrib of flag leaf blade were averaged, indicating the SPAD value for each plant. At least 8 plant individuals from each line were subjected to the investigation for SPAD value. Photosynthetic rate of flag leaves at 7 day after heading was measured using a portable photosynthesis system (LI-6400, LI-COR, Lincoln, USA) as described previously^57^. Photosynthesis rate measurement of at least 10 individuals from each line was conducted on clear days between 9 am and 11: 30 am. A digital plant stem strength detector (YYD-1A, TOP Instrument) was used to measure mechanical strength of the stems 30 days after heading, following the method as described previously^58^.

### Determination of pigment and total N of flag leaves

Fresh leaves were sampled to extract chlorophyll using the method described by Lichtenthaler^59^. Briefly, the fresh leaves whose main veins removed were cut into small pieces weighing approximately 50 mg, were homogenized, then were added into 8 ml 95% (v/v) alcohol. Pigments were extracted at 4 °C in dark. The measurements of chlorophyll *a*, chlorophyll *b*, total absolute chlorophyll, and carotinoid concentration were conducted with Spectrophotometers (DU640, Beckman Coulter) at 665 nm, 649 nm and 470 nm with 95% ethanol as blank control, respectively.

Leaf nitrogen content was determined in the method as described previously^60^. Leaf samples were inactivated at 105 °C for 30 min, then were dried at 80 °C until constant weight, and were ground to fine powders. The 0.2 g of dry powder was digested with 5 ml 98% H_2_SO_4_ (w/w) and 5 ml 30% H_2_O_2_ (w/w) in a conventional high temperature digester (AMS-Westoco, France). The digested sample was diluted with distilled water to 100 ml. The nitrogen concentration in the solution was determined on an automated discrete analyzer (SmartChem 200, France). All measurements were repeated three times.

### Measurement of starch, cellulose, and hemicellulose content

Flag leaves of main stem were collected, inactivated, ground through a 40 mesh sieve and stored in a dry container until use. Various cell wall components such as soluble sugar, lipid, starch, pectin, hemicellulose and cellulose were fractionated following the procedure described previously with minor modifications^61^. After sequentially extracted soluble sugar with potassium phosphate buffer (pH 7.0) and removed lipids with chloroform-methanol (1:1, v/v), starch were extracted using DMSO (dimethyl sulfoxide)–water (9:1, v/v). The remaining crude cell walls were treated with 0.5% (w/v) ammonium oxalate for pectin. The deposit was then suspended in 4 M KOH containing 1.0 mg ml^−1^ sodium borohydride, and the supernatants were neutralized and lyophilized as KOH-extractable hemicelluloses. The remaining residues were hydrolyzed trifluoroacetic acid (TFA), washed with distilled water and the supernatants were combined as the non-KOH-extractable hemicelluloses. The remaining pellets were defined as crude cellulose.

For soluble sugar determination, total hexoses and pentoses were measured in the potassium phosphate buffer fraction. For starch assay, it was estimated as total hexoses in the DMSO fraction by anthrone/H_2_SO_4_ assay^62^. For cellulose, the crude celluose fraction was dissolved in 67% H_2_SO_4_, and the supernatants were collected for determination of hexoses as cellulose^62^. For hemicelluloses assay, total hexoses and pentoses were measured in the hemicellulose fraction. The pentoses were detected by orcinol/HCl method^63^. The hexose and pentose measurements were performed using an UV/VIS Spectrometer (MAPADA, Shanghai). The experiments were performed three times.

### Monosaccharide and GlcA analysis of crude cell walls by GC-MS

Monosaccharide and GlcA analysis of crude cell walls was followed the method with minor modifications^64^. Briefly, after pectin removal as described above, the pellets were suspended in 2.5 ml desalted solution, and hydrolyzed with TFA (2 M) at 120 °C for 1 h. The supernatant was dried in vacuum, then was silanized, and diluted in 1.3 ml hexane for GC-MS analysis. GC/MS analysis was conducted with SHIMADZU GCMSQP 2010 Plus. D-glucuronic acid, D-xylose, D-arabinose, and D-glucose were used as GlcA or monosaccharide standards. Peaks were identified by mass profiles and/or retention times of standards.

### Hydroponic experiments

The 15-day-old seedlings were transferred to a standard nitrogen-deficiency rice culture solution described previously^65^, which was supplemented with 0.36 mM, 1.44 mM, and 2.88 mM NH_4_NO_3_ representing low-N, normal-N, and high-N conditions, respectively, and were grown for 3 weeks. Hydroponics experiments were carried out in a net room with 14-h light/10-h dark conditions with an average temperature of 26 °C. Plants were grown in a container with 50L nutrient solution for each treatment of low-N, normal-N, and high-N with two biological replicates. The nutrient solution was refreshed every 3 days. After 3-week growth, the plants were measured for SPAD-value, seedling length, and biomass.

### Observation of the cross-section of flag leaves

Central sections of flag leaf blades were sampled, fixed in 70% FAA, dehydrated through a series of ethanol gradient, and then embedded in paraffin wax. Leaves were cut using a rotary microtome (RM2235, Leica), and placed on lysine-treated slides, and dried for 2 days at 37 °C, de-waxed with xylene and hydrated through an ethanol gradient, stained with 0.05% toluidine blue, dehydrated with gradient alcohol and xylene dehydration, and then mounted as described previously^66^.

Vascular bundles of leaf mesophyll anatomy were observed under a fluorescence microscope (DM2000, Leica). The thickness of large and small vascular bundles and the distance between two consecutive small vascular bundles were measured and analyzed at 10-fold objective magnification with digital image processing software (ImagePro Plus v. 5.1, Mitani).

For the observation under transmission electron microscope, leaves at heading stage were cut into small pieces (1 mm^2^), fixed in 2.5% glutaraldehyde in a phosphate buffer (pH 7.2), vacuum infiltrated, rinsed, and incubated overnight at 4 °C in a solution of 1% OsO4. Samples were dehydrated in a series of ethanol, infiltrated in epoxy resin at 37 °C for 8–12 h, and embedded in Epon 812 resin at 60 °C for 48 h. A series of 80 nm sections were cut using an ultramicrotome (EM UC7, Germany), and observed in a transmission electron microscope (Tecnai G^2^ 20 TWIN, FEI, USA).

## Supplementary information

Supplemental information

## Data Availability

All data generated or analysed during this study are included in this published article and its Supplementary Information files.
