# Supplementary material for: A xylan glucuronosyltransferase gene exhibits pleiotropic effects on cellular composition and leaf development in rice
Source: Sci Rep. 2020 Feb 28;10:3726. doi: 10.1038/s41598-020-60593-3 (PMC7048734; doi:10.1038/s41598-020-60593-3)
Supplement: Supplementary file 1 — Supplemental information [file 41598_2020_60593_MOESM1_ESM.pdf]

A xylan glucuronosyltransferase gene exhibits pleiotropic effects on cellular composition and leaf development in rice

Dawei Gao[1], Wenqiang Sun[1], Dianwen Wang[1], Hualin Dong[1], Ran Zhang[2], Sibin Yu[1*]

[1]National Key Laboratory of Crop Genetic Improvement, College of Plant Science and Technology, Huazhong Agricultural University, Wuhan 430070, China
[2]Biomass & Bioenergy Research Centre, Huazhong Agricultural University, Wuhan 430070, China.

*Correspondence: ysb@mail.hzau.edu.cn

Supplemental Figures S1–11 and Tables S1–3

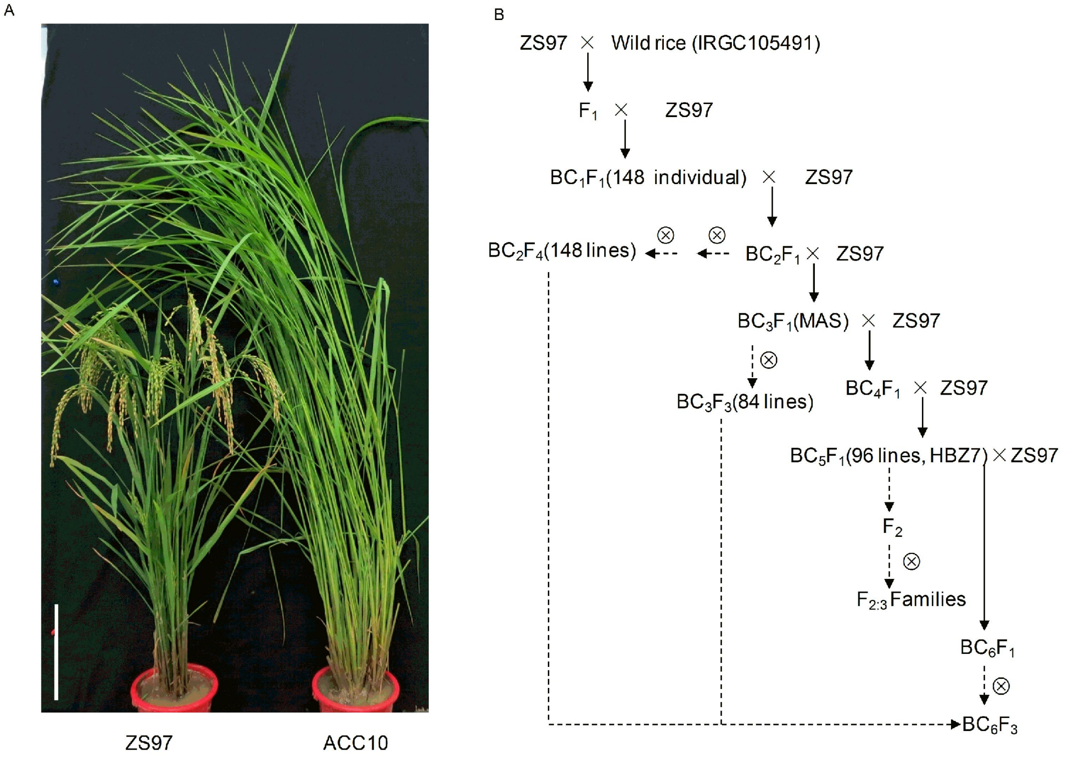

Fig. S1. Construction of the CSSL population from a cross of ZS97 and ACC10 by a backcross scheme.

(A) Performances of two parental lines ZS97 and wild rice ACC10. Bar = 20 cm.

(B) Development of chromosomal segment substitution lines (CSSLs) from the cross of *indica* rice ZS97 as recurrent and wild rice ACC10 as donor. One CSSL (HBZ7) that carried the target heterozygous segment was selected to self-cross to generate a segregating population for fine-mapping.

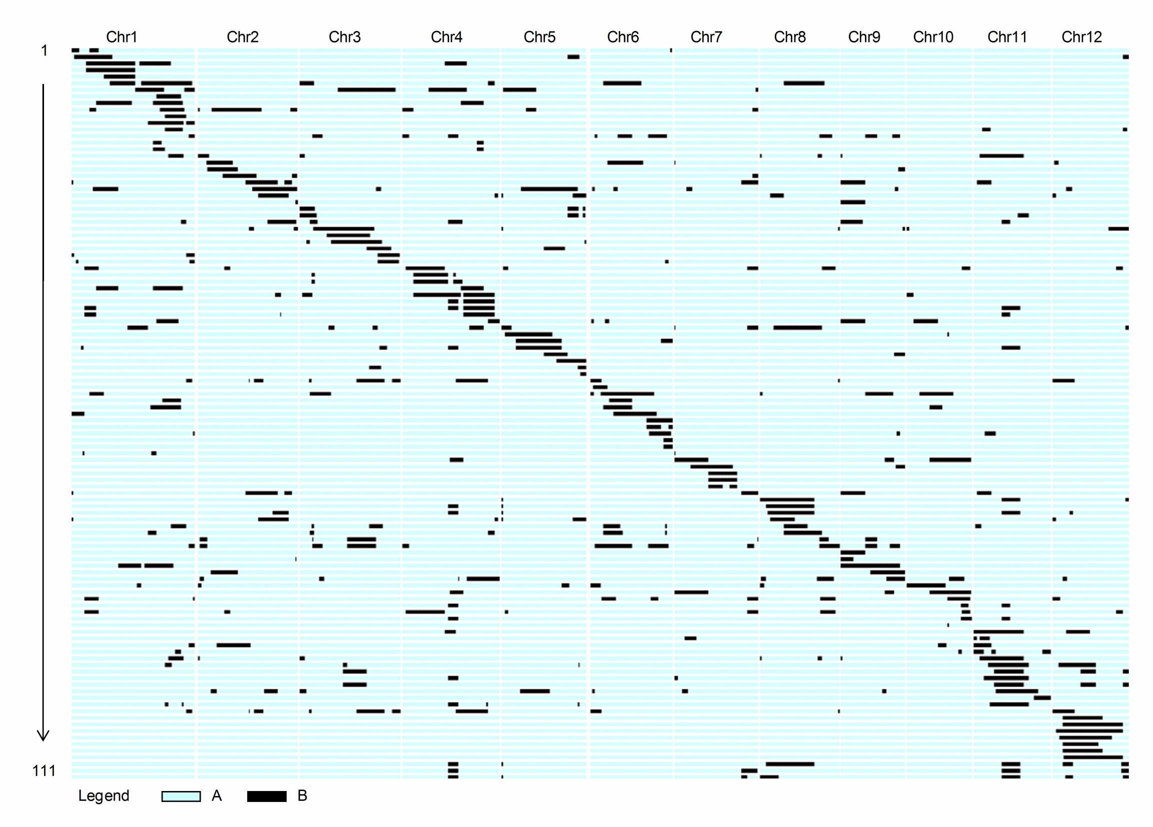

Fig. S2. Graphic genotype of the chromosome segment substitution lines.

The population is comprised of 111 lines and classed the whole genome into 379 Bins based on the RICE6K array. A (Light turquoise) and B (Black frame) represent ZS97 and ACC10 genotype, respectively.

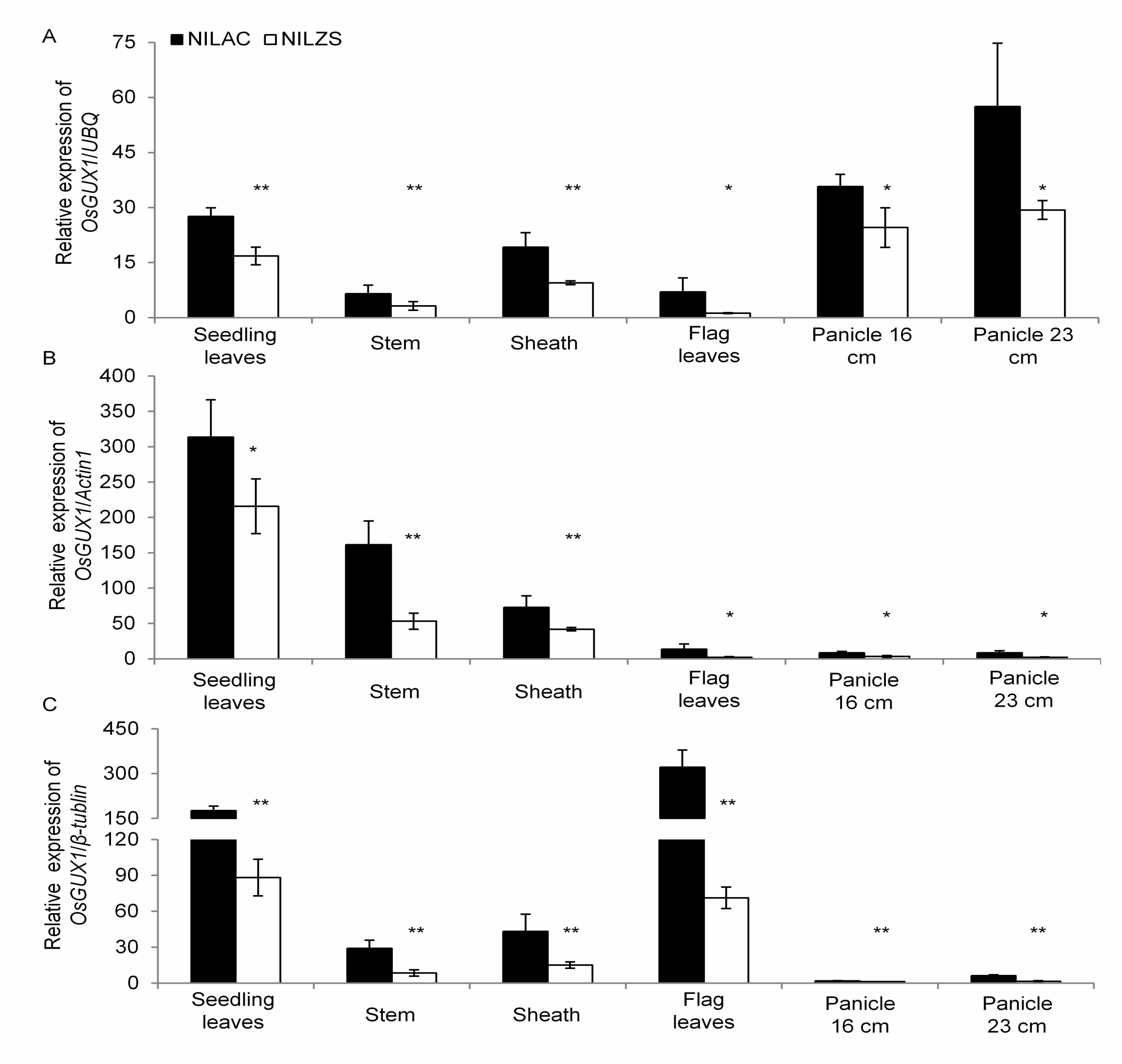

Fig. S3. Expression profile of *OsGUX1* in various tissues from the near isogenic lines (NILs) using three inference genes.

Relative expression of *OsGUX1* using (A)*Ubiquitin* (*UBQ*), (B) *Actin1*, (C) *β-tubulin* as the internal gene. The experiment with each sample was conducted in biological triplicates with four technical replicates. Data are presented as means ± SE (n = 3). The asterisks indicate significant differences between NIL^{AC} and NIL^{ZS} by student's t-test (*P<0.05, **P<0.01). This supplemental figure is related to Fig.2.

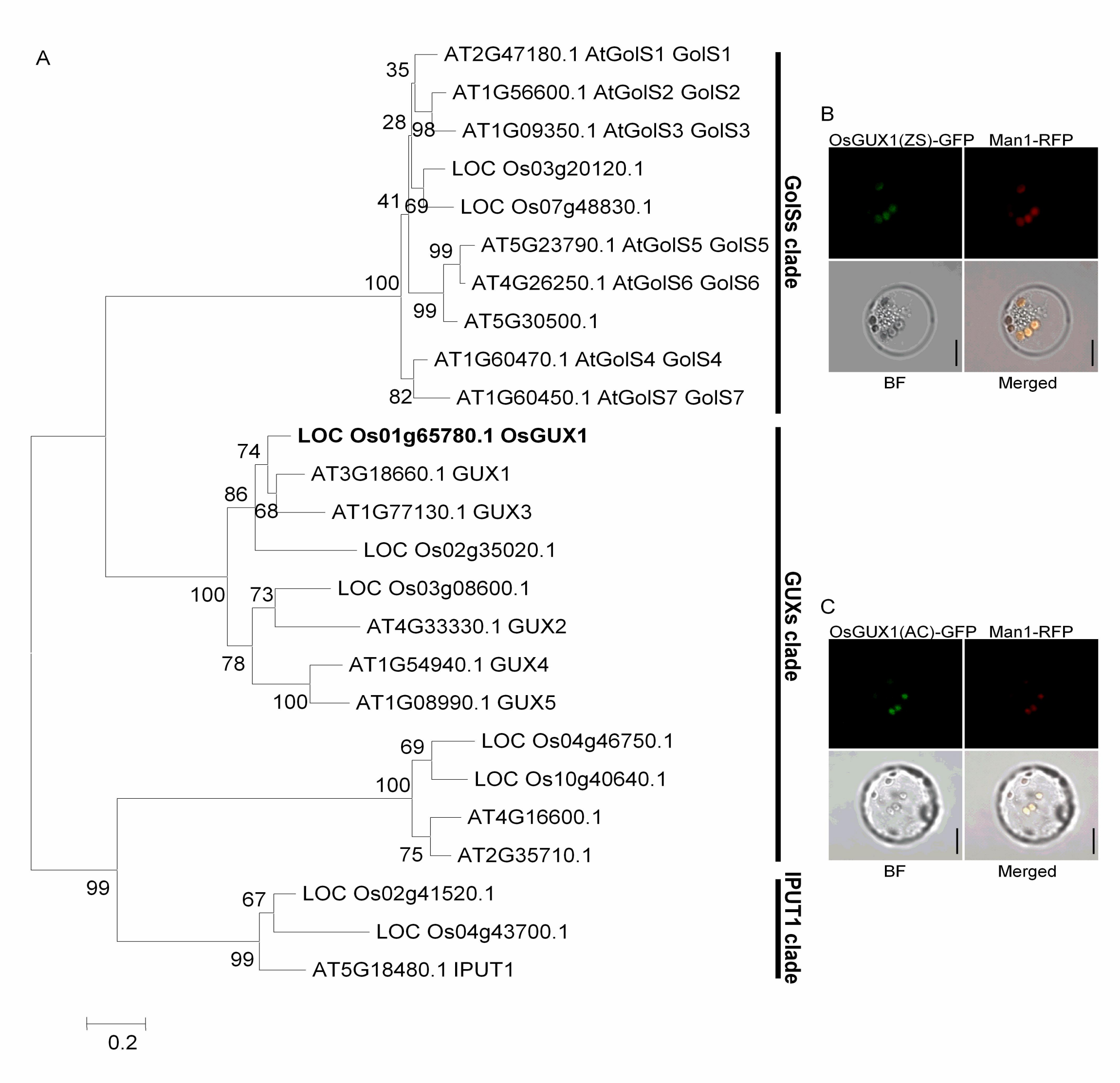

Fig. S4. Phylogenetic analysis and subcellular localization of *OsPGSIP1*.

(A) An unrooted phylogeny tree was constructed from amino acid sequences of the OsGUX1 and its homologs from *Oryza sativa* and *Arabidopsis thaliana* in Phytozome by using TBLASTP. Bootstrap values are based on 1000 replications and are indicated in their respective nodes. (B-C) Subcellular localization of OsGUX1^{ZS} and OsGUX1^{AC} protein in rice protoplasts, (B) OsGUX1^{ZS}-GFP co-localized with the Golgi marker Man1, (C) OsGUX^{AC}-GFP co-localized with the Man1. BF, bright field. Scale bar = 10 μm in (B, C).

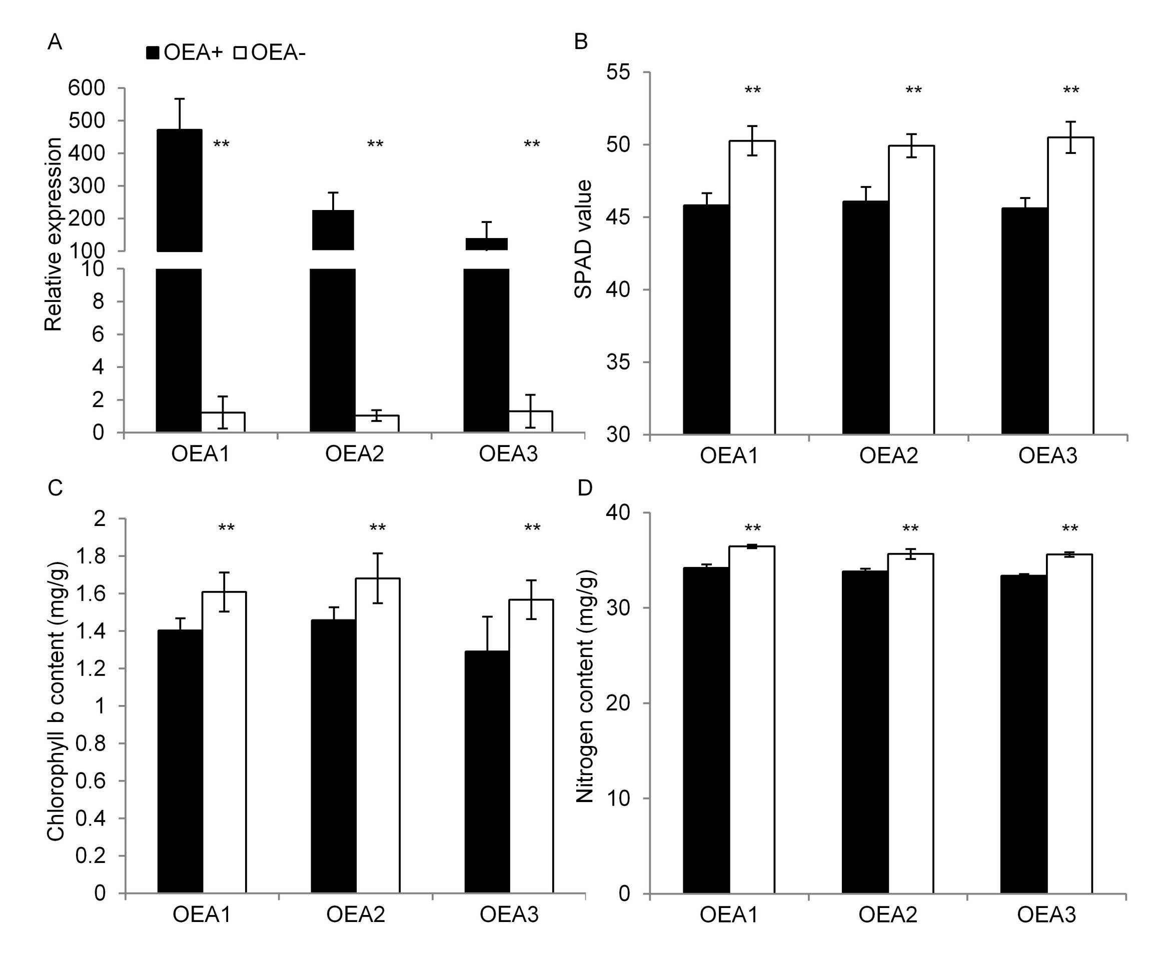

Fig. S5. Relative expression and nitrogen-related phenotype of flag leaves in three independent *OsGUX1^{AC}*-overexpression lines (OEA) on day 7 after heading.

(A) Relative expression of *OsGUX1^{AC}/Ubiquitin*. The error bar presents the mean ± SE (n = 3). (B) SPAD value. (C) Content of chlorophyll *b*. (D) Nitrogen content. Asterisks indicate the significant differences between the positive transgenic line (OEA+) and negative line (OEA-) by t-test (* $P < 0.05$, ** $P < 0.01$). The error bar presents the mean ± SE (n = 8).

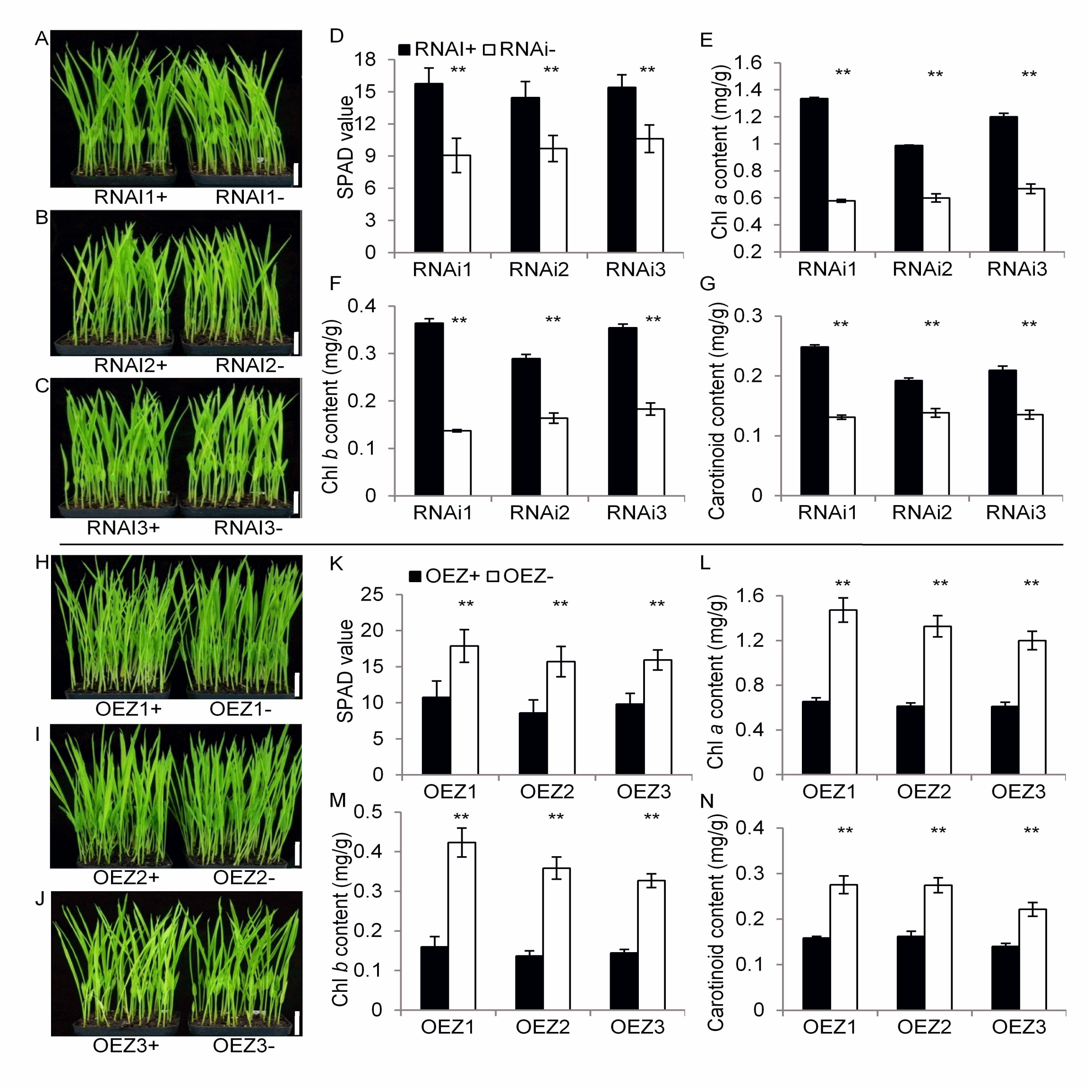

Fig. S6. Phenotypic characterization of *OsGUX1*^{ZS}-RNAi lines (RNAi) and overexpression lines (OEZ) at seedling stage.

(A-G) Performances of three dependent RNAi lines. (A-C)10-day-old seedlings, (D) SPAD value, (E) Contents of chlorophyll *a*, (F) chlorophyll *b* and (G)carotinoid.

(H-N) Performances of three dependent OEZ lines. (H-J)10-day-old seedling, (K) SPAD value, (L) Contents of chlorophyll *a*, (M) chlorophyll *b* and (N) carotinoid. Bars = 2 cm. Asterisks indicate the significant differences between the positive transgenic line (RNAi+, OEZ+) and negative line (RNA-, OEZ-) by t-test (* $P < 0.05$, ** $P < 0.01$). The error bar presents the mean ± SE (n = 8).

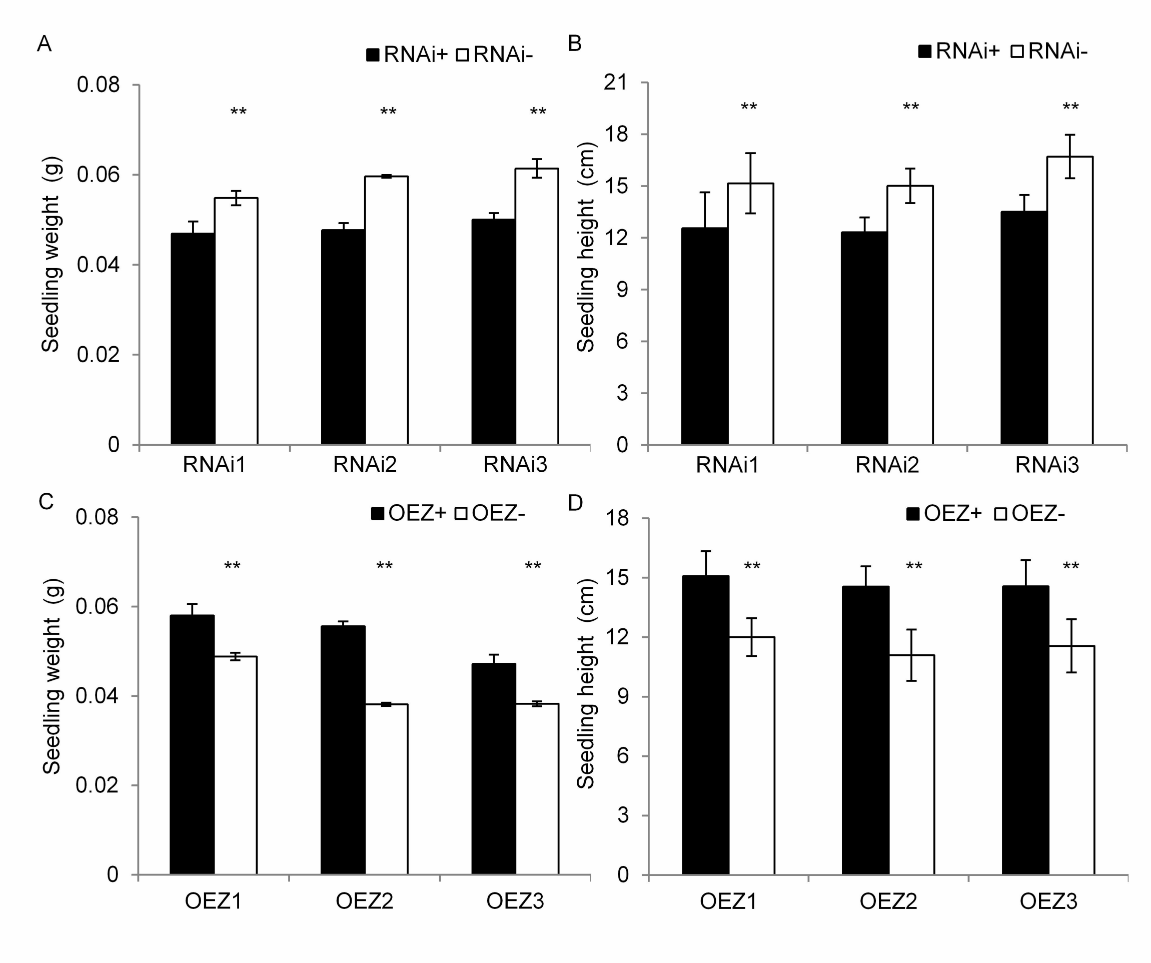

Fig. S7. Biomass of seedlings in *OsGUX1^{ZS}*-RNAi lines (RNAi) and overexpression lines (OEZ).

(A, C) seedling weight. (B, D) seedling height. Asterisks indicate the significant differences between the positive transgenic line (RNAi+, OEZ+) and negative line (RNA-, OEZ-) by t-test at $P < 0.01$. The error bar presents the mean ± SE (n = 3, with at least 15 seedling samples for each replicate).

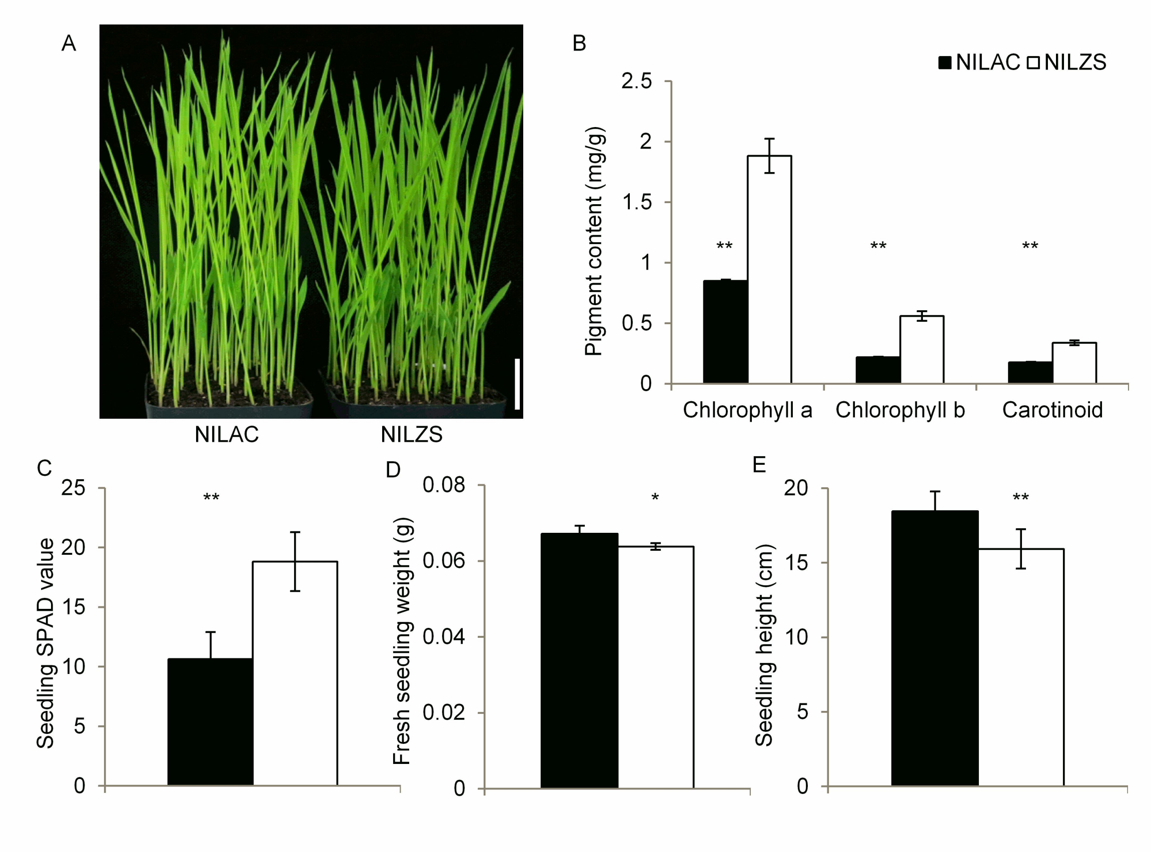

Fig. S8. Performances of near isogenic lines (NIL^{AC} and NIL^{ZS}) at seedling stage. (A) 14-day-old seedling. Bar = 2 cm. (B) Contents of chlorophyll a, chlorophyll b, and carotinoid. (C) SPAD value. (D) Fresh seedling weight. (E) Seedling height. Asterisks indicate the significant differences between the NILs by t-test (* $P < 0.05$, ** $P < 0.01$). The error bar presents the mean ± SE ($n = 3$, with at least 15 seedling samples for each replicate).

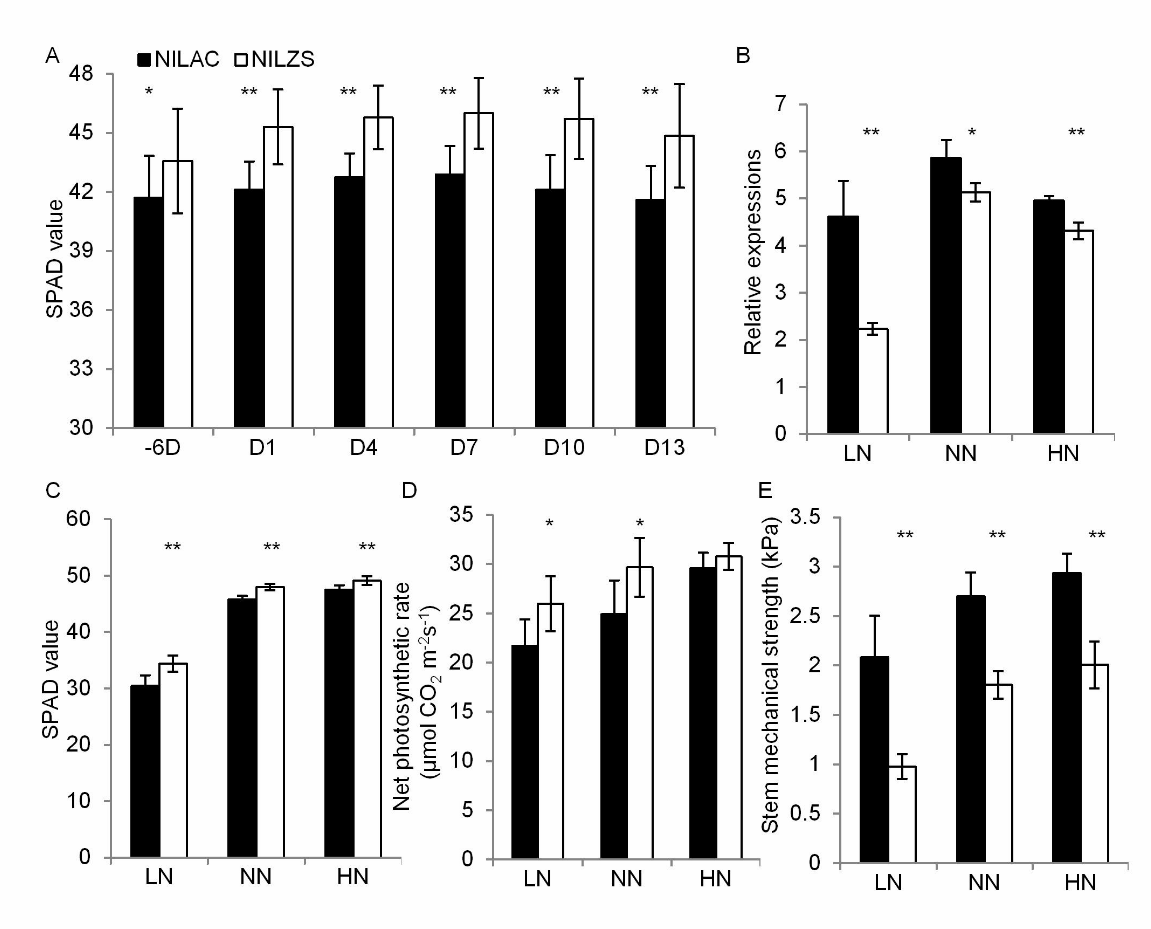

Fig. S9. Phenotype investigation and the relative expression of *OsGUX1* in the near isogenic lines (NILs) treated with different nitrogen application conditions.

(A) Dynamic investigation of SPAD value of flag leaves in NILs. -6D represents on day 6 before heading, D1, D4, D7, D10, and D13 represent day 1, day 4, day 7, day10, day13 after heading, respectively. (B) Relative expression of *OsGUX1* to *Ubiquitin* in NILs at seedling stage. (C) SPAD value of flag leaves in NILs on day 7 after heading. (D) Net photosynthetic rate of flag leaves in NILs. (E) Stem mechanical strength in NILs at maturity. Three nitrogen gradients LN, NN and HN, represent low, normal and high nitrogen application, respectively.The asterisks indicate significant differences between the NILs by t-test (*P<0.05, **P<0.01). The error bar presents the mean ± SE (n = 8-10 for SPAD measurement, n = 2 for other traits)

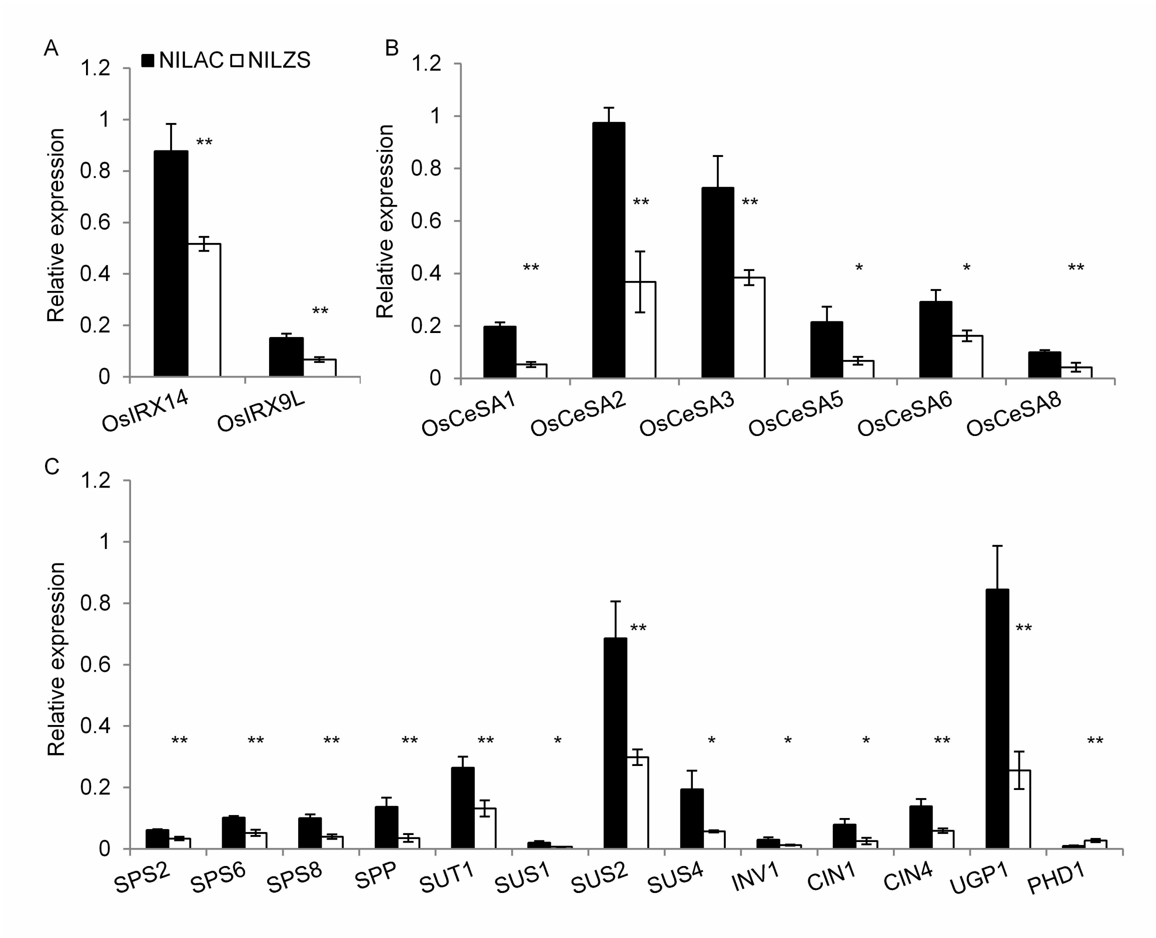

Fig. S10. Expression analysis of several key genes involved in carbohydrate metabolism and carbon allocation in flag leaves of NILs.

(A) Xylan synthesis-related genes. (B) Cellulose synthase-related genes. (C) Sucrose-related genes. The geometric average of the three control genes *(Ubiquitin, ACTIN1, β-tubulin)* for the relative expression analyses. *SPSs*, Sucrose Phosphate Synthases; *SPP*, Sucrose Phosphate Phosphatase; *SUT1*, Sucrose Transporter1; *SUSs*, Sucrose Synthases; *INV*, Invertase; *CIN*s, Cell wall Invertases; *UGP1,* UDP-Glc Pyrophosphorylase1; *PHD1*, Photoassimilate Defective 1. The asterisks indicate significant differences between the NILs by t-test (*P<0.05, **P<0.01)

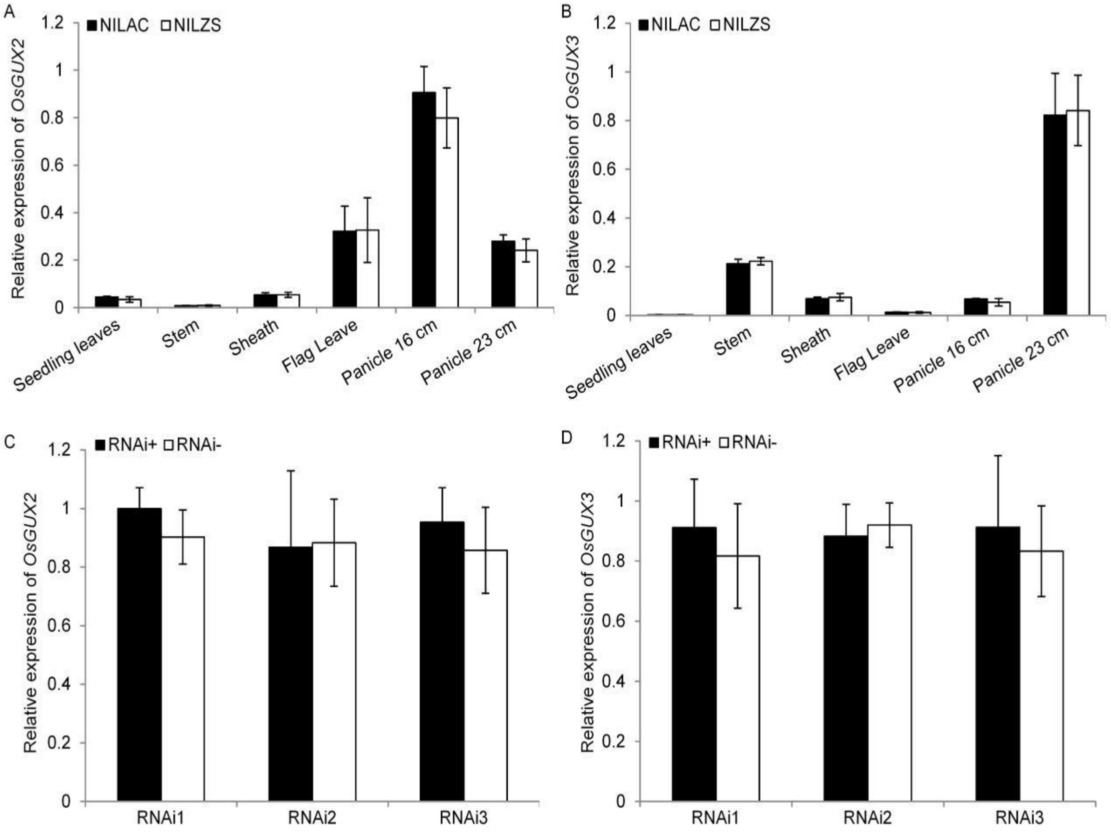

Fig. S11. Expression analyses of two close homologous genes of *OsGUX1*.

The expression levels of *OsGUX2* (A) and *OsGUX3* (B) relative to *Ubiquitin* in various tissues in NILs; The expression levels of *OsGUX2* (C) and *OsGUX3* (D) relative to *Ubiquitin* in leaf in the RNAi lines. The error bar presents the mean ± SE (n = 3 biological replicates). No significant differences observed in these paired lines.

Table S1. Descriptive statistics of agronomic traits of two parents and CSSLs

Trait	Parents		CSSLs		
	ACC10	ZS97	Range	AVE	CV (%)
Effective panicle	18.0±1.2**	13.0±0.9	6.8–16.9	11.9	18.3
Panicle length	27.8±0.4**	22.5±0.6	17.6–30.3	22.8	11.2
Primary branches	9.2±1.4	9.6±0.4	8.3–12.6	9.5	16.4
Full grain number	101.9±3.7*	115.3±4.3	43.1–192.8	87. 5	33.8
Grains per panicle	109.2±5.5*	116.6±8.0	73.2–232.6	118.9	25.0
KGW (g)	20.1±0.2**	28.1±0.2	20.7–33.7	26.3	5.9
Yield of plant (g)	27.8±2.3*	21.3±2.0	5.7–34.3	19.9	34.0
Heading date (days)	111.5±0.5**	75.5±2.5	54.5–118	68.6	13.6
Plant height (cm)	172.8±1.7**	86.1±1.2	56.2–166	89.7	22.1
SPAD value	33.8±0.4**	46.3±0.5	39.8–54.3	49.0	6.2

Note: CSSLs, chromosomal segment substitution lines; AVE, average of the population; CV, coefficient of variation; KGW, thousand grain weight; SPAD, Soil-Plant Analysis Development. The asterisks indicate significant differences between two parents by t-test (* $P < 0.05$, ** $P < 0.01$).

Table S2. QTLs for nitrogen content in the ACC10/ZS97 CSSL population

QTL	Chr	Bin	Bin size (kb)	LOD	PVE%	Add	Known gene
qNC1.1	1	35	75.0	3.04	4.82	-1.08	
qNC1.2	1	47	264.2	13.04	26.86	-2.67	
qNC7.1	7	257	413.3	5.84	9.95	-1.62	*Ghd7.1*
qNC10.1	10	313	94.1	12.05	24.16	-4.23	

Note: Quantitative trait loci (QTLs) analyses were performed by using the composite interval mapping method in IciMapping. LOD, logarithm of the odds; PVE%, phenotypic variance explained, Add, additive effect.

Table S3. Primers used in this study

Marker name	Primer 5'-3'	Application
SID01F	CTACCGTGTAAGCTCGTTGATTC	Fine-mapping
SID01R	CACCAATCAAACTAACAGCAACA	Fine-mapping
SID06F	GACCGATTTTTCCCACCTC	Fine-mapping
SID06R	GTGTTCGTTGTCCCTCTTCTATG	Fine-mapping
SPRD30F	TCTCACCGACTACGCTAATCAC	Sequencing
SPRD30R	AGGGAGCTGCAAAATCCAACAT	Sequencing
SPRD31F	ATCCTCCTCCCCTTCTTCCTCA	Sequencing
SPRD31R	ACCGCGTGTTCGTCGTCTCCAA	Sequencing
SPRD44F	TCTCAAGCAGCCAATCAATG	Sequencing
SPRD44R	GGCTAGCTTGGCATTAGCTG	Sequencing
SPRD45F	GATGGCCTTGGCTAGGAAAC	Sequencing
SPRD45R	GGCCTTGTTTGTGATTGTGA	Sequencing
SPRD46F	CAGTGTTGTCTTGCCACACC	Sequencing
SPRD46R	CGAAAGAGGGTCAAATAAGCA	Sequencing
SPRD47F	TCGAGTGTGGCCATGTTTTA	Sequencing
SPRD47R	CTGTGCACTACCGCTAATCG	Sequencing
SPRD48F	ACAAGGGCCTAGAGGACGAC	Sequencing
SPRD48R	CCAGGAGCAACACAAGAACA	Sequencing
SPRD49F	GGGAAATTGCCATCCCTAGT	Sequencing
SPRD49R	CCGCCATAGAGTTCTCATCC	Sequencing
SPRD32F	CTGAGACCGGAGAGCCATAG	Sequencing
SPRD32R	CTGGTGACCACTGAGGACTG	Sequencing
SPRD33F	CGGTGGTCATGTGTTTTCAC	Sequencing
SPRD33R	TGGAATTTGCACCAGGTATG	Sequencing
SPRD34F	GTCCATATAGGCCAGCCAGA	Sequencing
SPRD34R	ACAGCACATGGATACCACGA	Sequencing
SPRD35F	ACAATTGCTCCCGGATAGTG	Sequencing
SPRD35R	GATTCCGGATCCTTTGGATT	Sequencing
SPRD35L36F	GACAGACGGAGGGAAGCATA	Sequencing

SPRD35L36R	ATGTGCAGTTTGAAGGCTCA	Sequencing
SPRD36F	AAGTAACCACCACCGGAAGG	Sequencing
SPRD36R	CAGGGTTTTCCTTCGTTGAG	Sequencing
SPRD37F	GCAGGCTGAGAAAGCAAACT	Sequencing
SPRD37R	AATGGGGCTCATCACTTGAG	Sequencing
PU7801-2F	AAAGAGCTCCGTCGGTGCTCCCAAGT	cDNA detection
PU3R	AAACTGCAGCTCATGTTGCGTCACTCCAG	cDNA detection
35S780DWF	TCTAGAATGGGTTCTTTGGAGACGAC	Subcellular localization
35S780DWR	GTCGACTGAACTTGTGAGGCTCGCGG	Subcellular localization
PU7804F	AAAGAGCTCCTCGCAGAGAACGAAAGAGA GTCCC	Vetor overexpression
PU7804R	AAACTGCAGATCTGGTATAGGGCTATCTAC ACAT	Vetor overexpression
PDS780-1F	AAAGAGCTCGGATCCTGCTGACCCACCAAT CCTCTAT	Vetor RNAi
PDS780-1R	AAAACTAGTGGTACCACTTGTGAGGCTCGC GGTAGGT	Vetor RNAi
RT7801F	TGGATCCTGTGAACTTGCTG	Positive identification
RT7801R	TGGTCCATCAACAACTGGAA	Positive identification
PMCG1F	CTGCTCCACACATGTCCATT	Positive identification
PMCG1R	CCCACCATCTTGTGGAGCTA	Positive identification
PMCG2F	GGCTCACCAAACCTTAAACAA	Positive identification
PMCG2R	CTGAGCTACACATGCTCAGGTT	Positive identification
RealT780.2F	TGACCCACCAATCCTCTAT	Realtime
RealT780.2F	CACTCCAACCCAGCCTTTT	Realtime
SPS1F	CTGGGCAAGATCCCACCGACCGACT	Realtime
SPS1R	GTGGTTGATGTGCAGCAGGTAGTCC	Realtime
SPS2F	TGATAATGGGTAACCGTGAGGC	Realtime
SPS2R	CAGAGTGCTTATGATGCTTGGGAT	Realtime
SPS6F	CGTGGTAGAAAGAAGAGGGAGGA	Realtime
SPS6R	CAGAGCGTGACAACGGAGTGA	Realtime
SPS8F	GGAGATTCCTTGAGGGATGTTC	Realtime
SPS8R	CCATTTATTGCCTGTGGTAGCC	Realtime

SPPF	GTCTTGCGCTGAATGTAAAGCCTGA	Realtime
SPPR	TATCATCAAGTAAATTCATTCCGTG	Realtime
SUT1F	GCCAAGGAAGTTCCATTCAA	Realtime
SUT1R	TGAGGATCAGTTCCCTTTGG	Realtime
SUS1F	GCTTCCACATTGACCCATAC	Realtime
SUS1R	CTTGAGGGCATACAGCATCT	Realtime
SUS2F	GAGGCTGATGACCTTGACTGG	Realtime
SUS2R	CCCTCCATTACTTGGATGTGCT	Realtime
SUS4F	TCCGTGAACTGGCGAAGACT	Realtime
SUS4R	CCCAAGTTCGTCACTTGCTG	Realtime
INV1F	CACGACGCAGTGATCTGAGG	Realtime
INV1R	GATGAAACGCAGGGAATACG	Realtime
CIN1F	TACACGGTGGGCATCTACAA	Realtime
CIN1R	TCCAGCCATACCTITCTTGG	Realtime
CIN4F	CATGTTTGTGCCGGATACTG	Realtime
CIN4R	TGCCATCACCATCTAACCAA	Realtime
UGP1F	GCCAGAACAAACCCATCAAAC	Realtime
UGP1R	GTAACTCCAGAGCCGAACCAG	Realtime
PHD1F	AATGGCAGGAGGAACATCCA	Realtime
PHD1R	AGGCAGCTACTCTCACATCC	Realtime
IRX9LF	TTCTACCATCTTCGCGACATTA	Realtime
IRX9LR	GACTGGTCCTTGTAGTATCGTC	Realtime
IRX14F	GTCTGGTTAATCTGGTGGGTAT	Realtime
IRX14R	CCAGCTTCCATTTCCTTGATTC	Realtime
CesA1F	TTGACTTGCACGATCGATACG	Realtime
CesA1R	TCCCACATAAACTGGACCCTG	Realtime
CesA2F	TGTCACAGGCCCTGATACCC	Realtime
CesA2R	AAGCATTCCGGCAGCTAAGA	Realtime
CesA3F	CCGGATTGATCCTTTCTTAGCA	Realtime
CesA3R	GCTGAGCTACCACCATGGGAC	Realtime
CesA5F	TTCCTGGCAAAGAATGACGG	Realtime

CesA5R	AAATATGGGCACAAACACCTGC	Realtime
CesA6F	TGCGAAGAATAATGGCCCTC	Realtime
CesA6R	AAAACTTGGGCTTGATTCGC	Realtime
CesA8F	TGTTGAAGGTGCTGGATTCGA	Realtime
CesA8R	TGAGAGTGGAGGCAACAAACG	Realtime
UbqF	AACCAGCTGAGGCCCAAGA	Reference gene
UbqR	ACGATTGATTTAACCAGTCCATGA	Reference gene
ACTIN1F	ACATCGCCCTGGACTATGACCA	Reference gene
ACTIN1R	GTCGTACTCAGCCTTGGCAAT	Reference gene
β-tubulinF	GCTGACCACACCTAGCTTTGG	Reference gene
β-tubulinR	AGGGAACCTTAGGCAGCATGT	Reference gene
